# RegenHeart: A Time-Effective, Low-Concentration, Detergent-Based Method Aiming for Conservative Decellularization of the Whole Heart Organ

**DOI:** 10.1021/acsbiomaterials.0c00540

**Published:** 2020-08-20

**Authors:** Eleonora Dal Sasso, Roberta Menabò, Davide Agrillo, Giorgio Arrigoni, Cinzia Franchin, Chiara Giraudo, Andrea Filippi, Giulia Borile, Guido Ascione, Fabio Zanella, Assunta Fabozzo, Raffaella Motta, Filippo Romanato, Fabio Di Lisa, Laura Iop, Gino Gerosa

**Affiliations:** ‡ Cardiovascular Regenerative Medicine, Department of Cardiac Thoracic Vascular Sciences and Public Health, University of Padua, Padua 35128, Italy; § Institute of Neuroscience, National Research Council (CNR), Padua 35127, Italy; ⊥ Department of Biomedical Sciences, University of Padua, Padua 35122, Italy; ∥ Department of Medicine, University of Padua, Padua 35122, Italy; # L.I.F.E.L.A.B. Program, Consorzio per la Ricerca sanitaria (CORIS), Veneto Region, Padua 35128, Italy; ¶ Department of Physics and Astronomy ‘G. Galilei’, University of Padua, Padua 35122, Italy; □ Fondazione Bruno Kessler, Trento 38123, Italy; ○ Institute of Pediatric Research ‘Città della Speranza’, Padua 35127, Italy; △ Cardiac Surgery Unit, University Hospital of Padua, Padua 35128, Italy

**Keywords:** heart, decellularization, whole organ scaffold, whole heart regeneration

## Abstract

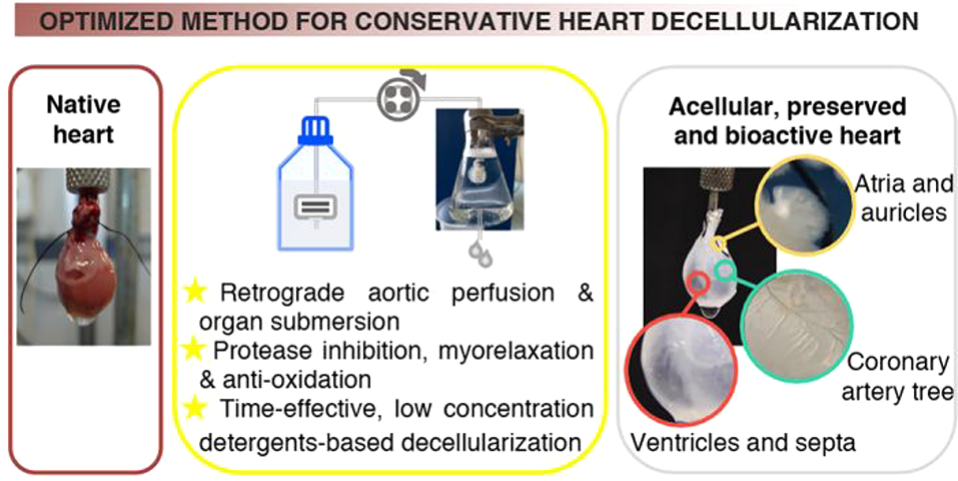

Heart failure is the worst outcome of all cardiovascular diseases and still represents nowadays the leading cause of mortality with no effective clinical treatments, apart from organ transplantation with allogeneic or artificial substitutes. Although applied as the gold standard, allogeneic heart transplantation cannot be considered a permanent clinical answer because of several drawbacks, as the side effects of administered immunosuppressive therapies. For the increasing number of heart failure patients, a biological cardiac substitute based on a decellularized organ and autologous cells might be the lifelong, biocompatible solution free from the need for immunosuppression regimen. A novel decellularization method is here proposed and tested on rat hearts in order to reduce the concentration and incubation time with cytotoxic detergents needed to render acellular these organs. By protease inhibition, antioxidation, and excitation–contraction uncoupling in simultaneous perfusion/submersion modality, a strongly limited exposure to detergents was sufficient to generate very well-preserved acellular hearts with unaltered extracellular matrix macro- and microarchitecture, as well as bioactivity.

## Introduction

Cardiovascular diseases continue to be the nightmare in the socio-ethical challenges of modern medicine. When not opportunely treated or, worse, if no efficacious therapy is available,^1−4^ diseases involving the myocardium, the coronary arterial tree, and/or heart valves evolve into heart failure, a clinical condition of cardiac dysfunction treatable only by transplantation with a natural or artificial replacement.^5,6^ Allogeneic heart transplantation remains the gold standard treatment for patients affected by severe cardiac failure. Despite public awareness campaigns and recently introduced regulations, organ donation is still limited by the number and quality of available allogeneic replacements; moreover, complications related to immunosuppressive therapies hamper the durability of surgical substitution.^7^ In an effort to overcome these limitations, artificial organs have been proposed as alternatives. Although several total artificial heart replacements have been conceived, only one has effectively reached clinical practice since 1980: no substantial evolutions were introduced, apart from the recent generation of a miniaturized version to be applied in pediatric and adult patients with a smaller stature.^8^ Yet, issues such as large size and low biocompatibility limit the clinical application to a restricted cohort of eligible patients.^6^

Starting in the 1990s, researchers in cardiovascular regenerative medicine began to propose novel strategies to rescue a failing heart. Approximately a decade ago, Ott and colleagues developed the first method for rodent heart decellularization by paving the way for the in vitro bioengineering of a functional cardiac replacement.^9^ The feasibility of decellularizing a whole heart opens the path to a more biocompatible replacement strategy based on a mature, specialized extracellular matrix (ECM), in which all biomolecules are distributed and organized as in the natural healthy organ. After cell removal, ECM and all its cell binding elements maintain the ability to guide repopulation and proper organization of the decellularized scaffold into a functional tissue or organ, as preclinically and clinically demonstrated in many approaches of tissue-guided regeneration and engineering.^10−13^ Besides removing cells, decellularization has also the aim of nullifying the immunogenic power by extracting allo- and xenoantigens from human and animal tissues.^10,14^ By combination with autologous or allogeneic cells, an approach based on such a scaffold has the potential advantage to give rise to a functional, self-like replacement therapy, devoid of the coadministration of debilitating immunosuppressive regimens.

Several techniques have been proposed so far for cell removal from rodent, porcine, and human hearts, based on different perfusion modalities and detergent cocktails, frequently potentiated in their action by physical and/or chemical agents.^15^ The method advanced by Ott et al. is a multistep decellularization with adenosine, heparin, 1% sodium dodecyl sulfate (SDS), 1% Triton X-100, and deionized water washes. The heart is retrogradely perfused at controlled pressure for a total of 13 hours, 12 of which with SDS, through the cannulated aorta in a modified Langendorff system.^9^ As in this pioneering work, several decellularizing protocols were developed by making use of different concentrations of SDS, an ionic detergent proven to be very effective in the removal of cells and their ECM-contacting proteins in comparative studies.^16−22^ SDS is, however, a very aggressive denaturing reagent, which can be detrimental for ECM. A working concentration equal or superior to 1% has been demonstrated to induce collagen and elastin precipitation as well as to damage the glycosaminoglycan moiety in treated cardiovascular structures of the heart,^23−28^ potentially leading to several mechanical dysfunctions, including valve incompetence and hence an inability to guarantee blood unidirectionality in the long term. Deleterious effects on the basal membrane have also been documented, thus compromising the potential of endothelialization. In the settings of the heart, the whole cell repopulation might be affected: apart from endothelial cells, cardiac myocytes also rely on a naturally meshed network of collagen IV, laminin, and heparan sulfate, and its damage may therefore have unfavorable consequences on the adhesion and/or expected working phenotype of repopulating cells.

An intact and biocompatible natural cardiac scaffold is, therefore, the first essential milestone in a successful approach of whole heart bioengineering.

In this study, we aimed to achieve an integral, acellular cardiac scaffold with preserved ECM and unmodified bioactivity, by developing an optimized and conservative perfusion decellularization method for the whole heart. In particular, we conceived a protocol based on limited exposure to a very low SDS concentration in combination with protease inhibitors and 2,3-butanedione monoxime, an excitation–contraction uncoupler, applied in an ex vivo experimental heart perfusion and suggested as a potential drug for cell death protection during cardiac ischemia.^29,30^

## Materials

### Reagents and Supplies

- Xilor (Bio 98 Srl, Milan, Italy)
- Zoletil (Virbac, Milan, Italy)
- Enoxaparin sodium (Clexane, Sanofi, Paris, France)
- Customized metal aortic cannula
- Polyvinyl calibrated peristaltic tubes (e.g., Gilson, Middleton, Wisconsin, USA, cat. no.: JGF117970)
- Sodium chloride (Sigma-Aldrich, Saint-Louis, MO, USA, cat. no.: S7653)
- Potassium chloride (Sigma-Aldrich, Saint-Louis, MO, USA, cat. no.: 793590)
- Sodium phosphate dibasic (Sigma-Aldrich, Saint-Louis, MO, USA, cat. no.: S5136)
- Potassium phosphate monobasic (Sigma-Aldrich, Saint-Louis, MO, USA, cat. no.: P5655)
- Phenylmethylsulfonyl fluoride (Sigma-Aldrich, Saint-Louis, MO, USA, cat. no.: P7626)
- N-ethylmaleimide (Sigma-Aldrich, Saint-Louis, MO, USA, cat. no.: E3876)
- Benzamidine hydrate (Sigma-Aldrich, Saint-Louis, MO, USA, cat. no.: B6506)
- Iodoacetamide (Sigma-Aldrich, Saint-Louis, MO, USA, cat. no.: I1149)
- Sodium ascorbate (Sigma-Aldrich, Saint-Louis, MO, USA, cat. no.: A7631)
- Ethylenediaminetetraacetic acid (EDTA, Sigma-Aldrich, Saint-Louis, MO, USA, cat. no.: E9884)
- Dimethyl sulfoxide (Sigma-Aldrich, Saint-Louis, MO, USA, cat. no.: D8418)
- Sodium dodecyl sulfate (SDS, Sigma-Aldrich, Saint-Louis, MO, USA, cat. no.: L3771)
- Triton X-100 (Sigma-Aldrich, Saint-Louis, MO, USA, cat. no.: X100)
- Benzonase (Sigma-Aldrich, Saint-Louis, MO, USA, cat. no.: E1014–25KU)
- 2,3-butanedione monoxime (Sigma-Aldrich, Saint-Louis, MO, USA, cat. no.: B0753)
- Penicillin-streptomycin (Sigma-Aldrich, Saint-Louis, MO, USA, cat. no.: P4333)
- Amphotericin B (Euroclone, Pero, Italy, cat. no.: ECM009D)
- Sucrose (Sigma-Aldrich, Saint-Louis, MO, USA, cat. no.: S7903)
- Paraformaldehyde (PanReac AppliChem, Darmstadt, Germany, cat. no.: A3813)
- Optimal cutting compound (OCT Tissue-TEK, Sakura, Japan, cat. no.: 4583)
- Haematoxylin-Eosin kit (e.g., Bio-optica, Milan, Italy, cat. no.: 04–061010)
- Masson trichrome kit (e.g., Bio-optica, Milan, Italy, cat. no.: 04–010802)
- Weigert/Van Gieson kit (e.g., Bio-optica, Milan, Italy, cat. no.: 04–053812)
- Alcian blue kit (e.g., Bio-optica, Milan, Italy, cat. no.: 04–160802)
- DNeasy blood and tissue kit (Qiagen, Venlo, Netherlands, cat. no.: 69504)
- Bovine serum albumin (Sigma-Aldrich, Saint-Louis, MO, USA, cat. no.: A2153)
- Anti-collagen IV antibody (rabbit polyclonal, Abcam, Cambridge, UK, cat. no.: ab6586)
- Anti-laminin antibody (rabbit polyclonal, DakoCytomation, Glostrup, Denmark, cat. no.: Z0097)
- Anti-rabbit goat immunoglobulins G secondary antibody, Rhodamine-conjugated (Millipore, Temecula, USA, cat. no.: AP132R)
- Hoechst (Sigma-Aldrich, Saint-Louis, MO, USA, cat. no.: 94403)
- Guanidine-hydrochloric acid (Sigma-Aldrich, Saint-Louis, MO, USA, cat. no.: G4505)
- Vivacon filters (cutoff: 10 kDa; Sartorius, Goettingen, Germany)
- Sodium acetate (Sigma-Aldrich, Saint-Louis, MO, USA, cat. no.: S2889)
- Chondroitinase ABC (Sigma-Aldrich, Saint-Louis, MO, USA, cat. no.: C3667)
- Keratanase (Sigma-Aldrich, Saint-Louis, MO, USA, cat. no.: G6920)
- Protease inhibitor cocktail (Sigma-Aldrich, Saint-Louis, MO, USA, cat. no.: P2714)
- Tris HCl (Sigma-Aldrich, Saint-Louis, MO, USA, cat. no.: T5941)
- Pierce BCA protein assay kit (Thermo Fisher Scientific, Waltham, MA, USA, cat. no.: 23225)
- Laemmli buffer (Sigma-Aldrich, Saint-Louis, MO, USA, cat. no.: S3401)
- Polyacrylamide gradient gels (NuPAGE, Invitrogen, Thermo Fisher, cat. no.: NP0326BOX)
- Colloidal Coomassie (Simplyblue Safestain, Invitrogen, cat. no.: LC6060)
- Ammonium bicarbonate (Sigma-Aldrich, Saint-Louis, MO, USA, cat. no.: A6141)
- Acetonitrile (Sigma-Aldrich, Saint-Louis, MO, USA, cat. no.: 34851)
- Dithiothreitol (Sigma-Aldrich, Saint-Louis, MO, USA, cat. no.: D9779)
- Sequencing grade modified trypsin (Promega Italia, Milan, Italy, cat. no.: V5111)
- Formic acid (Sigma-Aldrich, Saint-Louis, MO, USA, cat. no.: F0507)
- Barium sulfate (Prontobario HD, 98.45 g BaSO4/hg, Bracco Imaging Italia, Milan, Italy)
- Ultrasound gel (ECO SuperGel, Ceracarta, Forlì, Italy)
- Human bone marrow mesenchymal stem cells (PromoCell, Heidelberg, Germany, cat. no.: C12974)
- Biopsy punchers (Kai Industries, Tokyo, Japan, cat. no.: 26984)
- Polystyrene culture plastics (Corning, NY, USA, cat. no.: CLS3516)
- Cyanoacrylate glue (3 M Italia, Pioltello, Italy)
- Minimum essential medium-alpha modification (Sigma-Aldrich, Saint-Louis, MO, USA, cat. no.: M8042)
- Fetal bovine serum (FBS, Sigma-Aldrich, Saint-Louis, MO, USA, cat. no.: F2442)
- l-Glutamine (Sigma-Aldrich, Saint-Louis, MO, USA, cat. no.: G7513)
- Pierce LDH cytotoxicity assay kit (Thermo Scientific, cat. no.: 88953)
- CellTiter 96 aqueous solution cell proliferation assay (Promega Italia, Milan, Italy cat. no.: G5421)
- General: micropipettes, micropipette tips, electronic pipettors, serological pipettes, syringes and needles, laboratory glassware, plastic tubes, surgical scissors and forceps, nylon suture wire, histology grade glass slides, glass coverslips.

### Equipment

- Stereomicroscope (e.g., Zeiss, Milan, Italy, cat. no.: STEMI 1000)
- Peristaltic pumps (e.g., Minipuls 3, Gilson, Middleton, Wisconsin, USA, cat. no.: JGF155001)
- Cryostat (e.g., Leica Biosystems, Milan, Italy, cat. no.: CM 1850 UV)
- Light microscope (e.g., Olympus Italia SRL, Milan, Italy, cat. no.: CX43)
- Microscope camera (e.g., Nikon Eclipse 50i, Nikon Instruments Europe BV, Amsterdam, Netherlands, cat. no.: DS-i2)
- Microscope acquisition software (e.g., NIS-Elements D 3.2 software, Nikon Instruments Europe BV, Amsterdam, Netherlands, https://www.microscope.healthcare.nikon.com/it_EU/products/software/nis-elements)
- Nanodrop spectrophotometer (e.g., Thermo Scientific, Waltham, MA, USA, cat. no.: ND-2000)
- Mikro-Dismembrator (B. Braun Biotech International, Melsungen, Germany)
- Orbitrap XL mass spectrometer (Thermo Fisher Scientific)
- Nano high-performance liquid chromatography Ultimate 3000 (Dionex; Thermo Fisher Scientific, cat. no.: 6035.1942)
- Proteome Discoverer Software (version 1.4, Thermo Fisher Scientific, cat. no.: OPTON-30945)
- Skyscan 1172 Micro-CT (Skyscan, Bruker, Kontich, Belgium, cat. no.: Skyscan 1172)
- N-Recon software (Skyscan, Bruker, Kontich, Belgium, https://www.bruker.com/products/microtomography.html)
- Horos (Horosproject.org, sponsored by Nimble Co LLC d/b/a Purview, Annapolis, MD, USA, https://horosproject.org/about/)
- Light microscope (e.g., EVOS XL Core, Life Technologies, Monza, Italy, cat. no.: AMEX1000)
- Fluorescence microscope (e.g., Nikon Eclipse TE2000-U, Nikon Instruments Europe BV, Amsterdam, Netherlands, cat. no.: TE2000-U)
- Spectrophotometer (e.g., FLUOstar Omega, BMG LABTECH, Ortenberg, Germany, cat. no.: FLUOstar)
- General: electronic balance, pH meter, planar shaker, tube rotator, cell incubator (37 °C and 5% CO_2_), BSL2-rated biosafety cabinet, freezer (−20 °C and −80 °C), refrigerated centrifuge for 1.5–2 mL tubes (e.g., Hettich, Tuttlingen, Germany, cat. no.: MIKRO 220R), refrigerated centrifuge for 15–50 mL tubes (e.g., Eppendorf, Milan, Italy, cat. no.: 5804R), cell counter chamber (e.g., Neubauer chamber, Saint-Louis, MO, USA, cat. no.: BR717810).

## Procedure

### Overview

- Step 1: Heart harvesting
- Step 2: Whole heart decellularization
- Step 3: Histological evaluation
- Step 4: DNA quantification
- Step 5: Multiphoton microscopy analyses
- Step 6: Proteomic analyses
- Step 7: Microcomputed tomography imaging
- Step 8: Cytocompatibility analyses

#### Step 1: Heart Harvesting

All experimental procedures were performed following the European Directive 2010/63/EU and Italian law 26 (04/03/2014) on animal-based research. In the present case, hearts were obtained as discard from euthanized animals in another ethically approved, experimental study (Project 87/2011 authorized by Italian Ministry of Health, IACUC equivalent).

1. Anesthetize each rat (Wistar breed, 150 g; male) with a subcutaneous injection of Xilor (0.4 mg/100 g) and Zoletil (9 mg/100 g) after enoxaparin sodium administration (200 UI/150 g).
2. After median sternotomy, isolate the heart of each animal and maintain it in a solution of ice-cold phosphate buffer saline (PBS), until further processing.
3. Place the heart under a stereomicroscope and gently remove surrounding unnecessary tissues (e.g., thymus, lungs, pericardium, and retrosternal fat).
4. Cannulate the ascending aorta using the customized cannula.

#### Step 2: Whole Heart Decellularization

A modified Langendorff apparatus equipped with a peristaltic pump is used to perfuse the hearts with the decellularizing solutions through the cannulated aorta, as previously reported.^15,31^ This circuit achieves the retrograde perfusion down the aorta, the opposite direction from its physiologic blood flow. With a closed aortic valve, the coronary arteries are perfused through their ostia in Valsalva’s sinuses. Fluids and cellular debris are then drained by the coronary veins into the coronary sinus. All solutions shall be prepared shortly before use.

1. Prior to the actual decellularization process, perfuse the heart with a solution of PBS, pH 7.4, added to 5 UI/mL enoxaparin sodium to wash the coronary arterial tree from blood residues (peristaltic pump speed 10 mL/min for 5 min followed by 40 min at 1.5 mL/min, + 4 °C).
2. After this first wash, place the cannulated heart in a glass reservoir in order to perform the decellularization by simultaneous perfusion and submersion of the organ in the decellularization solutions.
3. Decellularize the heart following these steps:
  a. Protease activity inhibition (30 min, pump speed 1.5 mL/min, room temperature (RT)): dilute 2 mM phenylmethylsulfonyl fluoride, 5 mM N-ethylmaleimide, 5 mM benzamidine, and 1 mM iodoacetamide in an antioxidant solution made of 10 mM sodium ascorbate and 5 mM EDTA in PBS plus 10% dimethyl sulfoxide.
  b. Antioxidation (30 min, 1.5 mL/min, RT): perfuse the heart with the antioxidant solution described in a.
  c. SDS-based perfusion (5.5 h, 10 mL/min, RT): perfuse 0.5% SDS in deionized water for heart decellularization.
  d. Wash in deionized water (30 min, 1.5 mL/min, RT).
  e. Triton X100-based perfusion (1 h, 1.5 mL/min, RT): perfuse 1% Triton X100 to facilitate the removal of SDS residues.
  f. Wash in PBS (overnight, 1.5 mL/min, RT).
  g. Benzonase-based treatment (1500 U/cm^2^; 2 × 24 h incubation, mild agitation, 37 °C): remove the heart from the Langendorff apparatus and incubate it in a solution of nonspecific nucleases to digest the nucleic acids.^10^
    **Please note that 2,3-butanedione monoxime (20 mM) was added at each step starting from the first PBS washout until SDS-based treatment to induce muscle relaxation and optimize whole organ perfusion.**
4. Maintain the decellularized heart in PBS supplemented with 3% penicillin-streptomycin and 0.25% amphotericin B and store it at +4 °C until further processing.

#### Step 3: Histological Evaluation

1. Perfuse native and decellularized hearts (*n* = 3 each) with 2% paraformaldehyde for 1 h at 10 mL/min.
2. Embed heart samples in a 1:1 solution of 20% sucrose and OCT and freeze under liquid nitrogen fumes.
3. Prepare cryosections of decellularized heart samples (8 μm thickness) with a cryostat.
4. Perform Haematoxylin-Eosin, Masson trichrome, Weigert/Van Gieson, and Alcian blue staining.
5. Acquire the images by means of a light microscope equipped with a camera.

#### Step 4: DNA Quantification

1. Freeze native and decellularized hearts (*n* = 6 each) by means of liquid nitrogen fumes.
2. Sample each heart, weigh the isolated tissue, and extract the DNA using the DNeasy blood and tissue kit as per the manufacturer’s instructions.
3. Quantify the extracted DNA using the Nanodrop spectrophotometer and normalize per tissue weight.

#### Step 5: Multiphoton Microscopy Analyses

These analyses are performed combining techniques of immunofluorescence and multiphoton microscopy using an in-house two-photon microscope (TPM), acquiring the second harmonic generation (SHG) and two-photon emitted fluorescence, as recently described.^32^ Use *n* = 3 samples for each group of native and decellularized hearts.

1. Assess the general histoarchitecture acquiring collagen and elastin signals on label-free cryosections, obtained as described in Step 3 (phases 1–3).
2. In order to assess the basal membrane integrity, perform an indirect immunofluorescence coupled with TPM acquisition, following these steps:
  a. Wash the cryosections obtained as described in Step 3 (phases 1–3) in PBS for 5 min in order to dissolve the OCT.
  b. Dilute anti-collagen IV (1:100) and anti-laminin (1:100) primary antibodies in a solution of 1% bovine serum albumin in PBS.
  c. Distribute the primary antibodies on the appropriate tissue sections and incubate for 1 h at 37 °C.
  d. Perform 2 washes in PBS for 5 and 10 min, respectively, at RT.
  e. Dilute the secondary anti-rabbit antibody (1:100) in a solution of 1% bovine serum albumin in PBS.
  f. Distribute the secondary antibody appropriately and incubate for 30 min at 37 °C.
  g. Perform 2 washes in PBS for 5 and 10 min, respectively, at RT in the dark.
  h. Prepare a counterstaining Hoechst solution (1:10000) in PBS in a glass jar.
  i. Incubate the sections in Hoechst solution for 5 min, at RT and in the dark.
  j. Perform 2 washes in PBS for 5 and 10 min, respectively, at room temperature and in the dark.
  k. Acquire the images using the TPM, as described by Zouhair et al.^33^

#### Step 6: Proteomic Analyses

Use a proteomic approach to characterize the protein composition of the decellularized hearts (*n* = 3), following an adapted method with respect to the one previously developed by Didangelos et al.^34^

1. Homogenize the decellularized cardiac samples (30–50 mg from a pool of different animals) with Mikro-Dismembrator.
2. Extract the proteins with a guanidine-hydrochloric acid (HCl) buffer (4 M guanidine HCl, 50 mM sodium acetate, 25 mM EDTA, pH 5.8) for 48 h in constant stirring at RT.
3. Centrifuge the samples at 10 .000 *g* for 10 min at +4 °C.
4. Add a protease inhibitor cocktail during extraction.
5. Remove possible contaminants applying the filter-aided sample preparation method by means of Vivacon filters and serial centrifugations at 14 000 *g* for 10 min at RT in the presence of extraction buffer.
6. Wash the extracted proteins with a pH 6.8 buffer composed of 150 mM sodium chloride and 50 mM sodium acetate, supplemented with protease inhibitor mixture.
7. Solubilize proteoglycans with a deglycosylation buffer (0.05 unit of chondroitinase ABC and 0.05 unit of keratanase in 150 mM sodium chloride, 50 mM sodium acetate, and protease inhibitors) for 16 h at +37 °C, followed by centrifugation at 14 000 *g* for 10 min at +4 °C.
8. Wash the deglycosylated samples with 60 mM Tris HCl buffer, pH 6.8, and centrifuge at 14 000 g for 10 min at +4 °C.
9. Measure protein concentration through the bicinchoninic acid method by means of a Pierce BCA protein assay kit and spectrophotometric reading at 562 nm.
10. Perform denaturation and reduction in Laemmli buffer at +96 °C for 10 min.
11. Carry out the electrophoresis in 4–12% polyacrylamide gradient gels at 30 mA constant current and stop as soon as all proteins have entered the gel and are packed in a single band of about 1 cm.
12. Stain the gel with colloidal Coomassie.
13. Extensively destain the gel with water, excise the protein bands, and cut then into 1 mm^3^ cubes.
14. Repeatedly treat the gel pieces with water and a solution of 200 mM ammonium bicarbonate/acetonitrile (60%/40% v/v).
15. Dehydrate the gel pieces with two 10 min washes of acetonitrile and dry under a vacuum.
16. Swell the dried gel pieces in 10 mM dithiothreitol in 50 mM ammonium bicarbonate and incubate for 1 h at 56 °C.
17. Alkylate the cysteine residues by treating the samples with 55 mM iodoacetamide in 50 mM ammonium bicarbonate for 45 min in the dark and at RT.
18. Wash sequentially the gel pieces with 50 mM ammonium bicarbonate (10 min) and acetonitrile (10 min) and dry under vacuum.
19. Treat the gel pieces with 40 μL of 50 mM ammonium bicarbonate containing 12.5 ng/μL sequencing grade modified trypsin. Incubate the samples overnight at 37 °C.
20. Extract the peptides with three changes of 50% acetonitrile/0.1% formic acid.
21. Dry the peptide mixtures under vacuum.
22. Dissolve the samples in 50 μL of 3% acetonitrile/0.1% formic acid.
23. Use 5 μL of each dissolved sample for liquid chromatography tandem mass spectrometry (LC-MS/MS) analysis, performed on a linear trap quadrupole (LTQ) Orbitrap XL mass spectrometer coupled online with a nano high-performance liquid chromatography (HPLC).
24. Separate the peptides using a linear gradient of acetonitrile from 3 to 40% in 40 min.
25. Operate the instrument in a data-dependent mode by performing a full MS scan (300–1700 *m*/*z* range) at high resolution (60000) in the Orbitrap, followed by fragmentation in the linear ion trap on the 10 most relatively abundant ions.
26. Analyze the raw data files with the software Proteome Discoverer coupled with a Mascot search engine (version 2.2.4, Matrix Science) against the rat section of the Uniprot database (version 20150401, 29382 sequences). Set carbamidomethylation of cysteines and methionine oxidation as fixed and variable modifications, respectively. Set trypsin as digesting enzyme with up to 1 missed cleavage allowed. Protein and peptide mass tolerance are established as 10 ppm and 0.6 Da, respectively.
27. Use the algorithm Percolator to assess the false discovery rate (FDR) with a parallel search against a randomized database.
28. Filter the results with FDR ≤ 0.01 and proteins are considered as positive hits if at least 2 unique peptides per protein are identified.
29. Group proteins into protein families following the principle of maximum parsimony.

#### Step 7: Microcomputed Tomography Imaging

1. Fill the coronary arteries of the decellularized hearts (*n* = 3) with a solution composed of 1 mg of barium sulfate, 2 mL of water, and 1 mL of ultrasound gel by means of a small-size introducer sheath.
2. Place the heart in vertical position into a cylindrical polyethylene container (1.1 cm diameter).
3. Scan each sample using a microcomputed tomography Skyscan scanner with the following settings: 63 kV of voltage; 157 μA of current; 1 mm aluminum filter; a 1280 × 1024 pixel field of view; 17 μm isotropic voxel size. The samples shall undergo a 360° rotation, with a step of 0.4° and a frame averaging of two.
4. Reconstruct the acquired raw data with the N-Recon software using the back-projection algorithm to reconstruct axial subsequent images saved as bitmap format.
5. Convert the acquired bitmap images into DICOM.
6. Perform the 3D reconstructions using Horos.

#### Step 8: Cytocompatibility Analyses

Evaluate the cytocompatibility degree of decellularized rat hearts in accordance with ISO 10993-5^35^ with human bone marrow mesenchymal stem cells (hBM-MSCs; PromoCell, Heidelberg, Germany).

1. Cut round patches of decellularized heart ventricles (DVs) by using biopsy punchers (area= 0.79 cm^2^).
2. Decontaminate obtained samples, as previously described by Fidalgo et al.^36^
3. Incubate so-treated DVs with FBS at 37 °C for 12 h followed by human fibronectin (5 μg/cm^2^) for 8 h.^10^
4. Perform direct contact assays and static seeding of preconditioned DVs (*n* = 6 each) with hBM-MSCs (p7; seeding densities: 0.015 × 10^6^ cells/cm^2^ for each). Consider cells seeded onto polystyrene culture plastics or in the presence of cytotoxic cyanoacrylate glue as controls. Use minimum essential medium-alpha modification, added with 20% FBS, 1% l-glutamine and 1% penicillin-streptomycin as culture medium.
5. Monitor cell phenotype, cytotoxicity and proliferation at several time points, i.e., 1, 2, 3, 7, 10, and/or 14 days in all conditions. Acquire images of cell morphology and distribution with a microscope, equipped with camera and acquisition software.
6. Quantify the lactate dehydrogenase (LDH) release, a marker of cell death, in cell media by using Pierce LDH cytotoxicity assay kit, according to the manufacturer’s instructions, and a plate reader spectrophotometer (wavelength: 490 nm). Express the quantification as percentage of cytotoxicity, as previously reported by Iop et al.^12^
7. Measure cell proliferation activity by means of a colorimetric MTS test (following the manufacturer’s instructions), based on the ability of viable cells to reduce tetrazolium salts in formazan dye. Use a plate reader spectrophotometer (wavelength: 490 nm) to measure absorbance.

### Timing

#### Step 1: Heart Harvesting

- Animal anesthesia and euthanasia: approximately 30 min
- Heart isolation and cannulation: 10 min

#### Step 2: Whole Heart Decellularization

- Initial wash: 45 min
- Protease activity inhibition: 30 min
- Antioxidation: 30 min
- SDS-based perfusion: 5.5 h
- Wash in deionized water: 30 min
- Triton X-100-based perfusion: 1 h
- Wash in PBS: overnight
- Benzonase-based treatment: 48 h

#### Step 3: Histological Analysis

- Haematoxylin-Eosin staining: 3 min
- Masson trichrome staining: 35 min
- Weigert/Van Gieson staining: 1 h 20 min
- Alcian blue staining: 50 min

#### Step 4: DNA Quantification

- DNeasy blood and tissue kit-based extraction: about 13 h

#### Step 5: Multiphoton Microscopy Analyses

- Indirect immunofluorescence: about 2 h

#### Step 6: Proteomic Analysis

- Whole analysis: 2 days

#### Step 7: Microcomputed Tomography Imaging

- Image acquisition: 5–6 h

#### Step 8: Cytocompatibility Analyses

- Cytocompatibility assessment: 14 days
- LDH cytotoxicity assay: 30 min
- MTS test: 3 h

### Troubleshooting

#### Step 1: Heart Harvesting

- Aorta should be kept as long as possible in order to facilitate cannulation.

#### Step 2: Whole Heart Decellularization

- Use a Langendorff system to perfuse the heart^15^ and a capable recipient for its submersion.
- In order to avoid the formation of air bubbles into the heart during the decellularization process, carefully check the Langendorff circuit and prevent the entrance of air through the solution reservoir. If needed, an air trap can be inserted in the circuit.

#### Step 4: DNA Quantification

- In case of need, prolong enzymatic incubation to ensure complete tissue digestion and effective DNA extraction.
- To maximize DNA extraction, the elution step can be repeated twice without adding further buffer, as follows: collect the centrifuged eluted volume and pipet it again directly onto the DNeasy membrane, incubate for 1 min and then centrifuge again.

#### Step 6: Proteomic Analyses

- Carefully mince and digest heart tissues.
- Pool more samples if the protein concentration is too low.
- Use centrifuge filters to increase the quality of protein extraction.

#### Step 7: Microcomputed Tomography Imaging

- Suspend each heart into the specimen holder in order to prevent wall collapse during scanning.
- In order to perform a ventriculography, force the aortic valve to its open position and fill the ventricle chambers with the barium-based contrasting medium before scanning.

### Anticipated Results

#### Steps 1–4: Rat Hearts Were Completely Acellular after Decellularization

**Figure 1:**
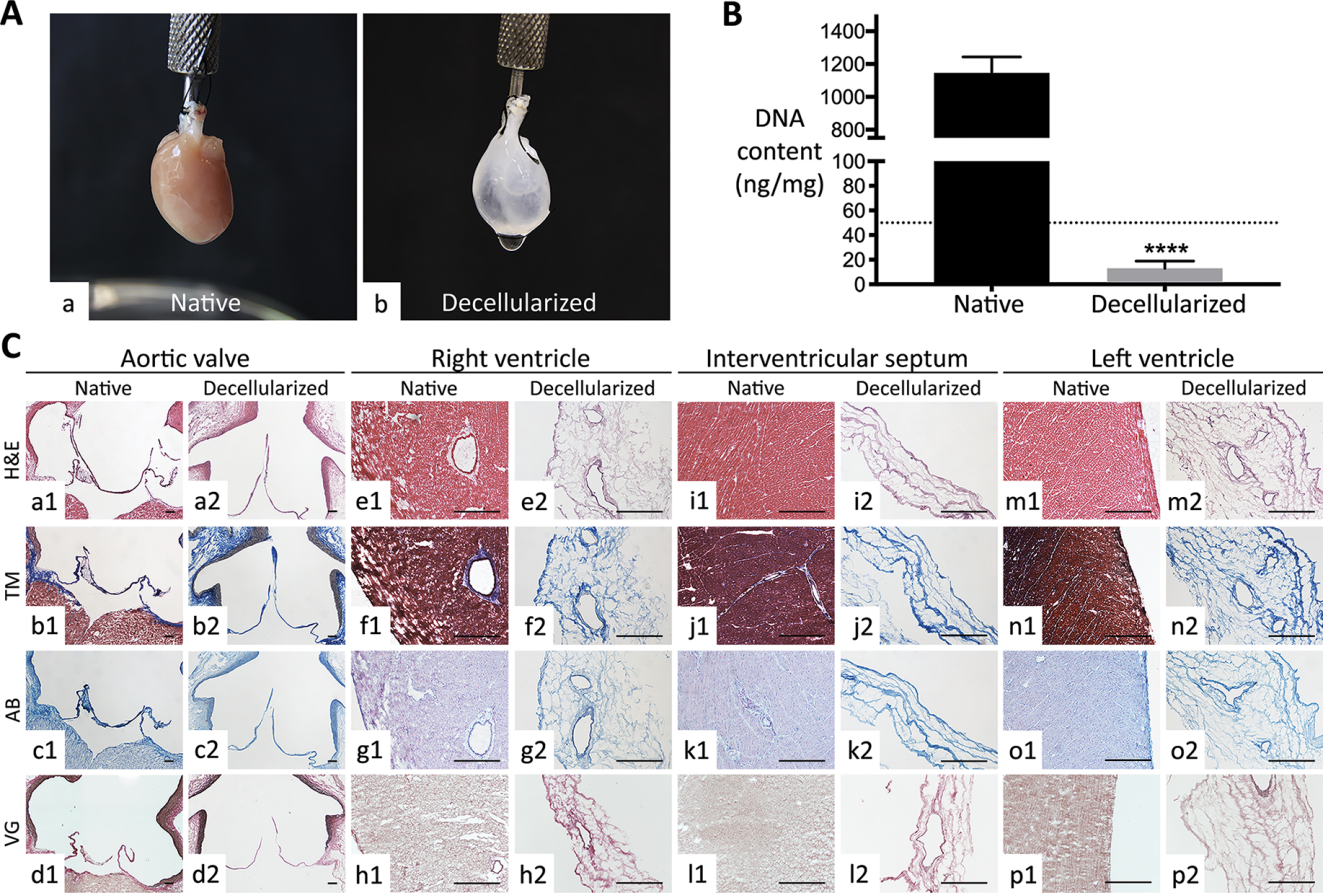
Decellularization yield of whole rat hearts. (A) Macroscopic view of (a) native and (b) decellularized hearts. Note the discoloration and the natural ECM scaffold (auricles, interventricular septum, coronary arterial tree) rendered visible at naked eye after decellularization. (B) DNA quantification pre- and postdecellularization. Decellularized hearts possessed a significantly lower DNA content with respect to native ones. Data are expressed as media ± standard deviation (****, *p* < 0.0001). The value of DNA residue for treated hearts was under the threshold of 50 ng/mg (dotted line) defined for decellularization by Crapo et al.^37^ (C) Histological evaluation of organ architecture pre- and post-decellularization. Valve apparatus (e.g., aortic valve), right and left ventricles, and interventricular septum of decellularized hearts showed a conserved ECM at Haematoxylin-Eosin (H&E; a2, e2, i2, and m2), Masson trichrome (MT; b2, f2, j2, and n2), Alcian Blue (AB; c2, g2, k2, and o2), and elastic Van Gieson (VG; d2, h2, l2, and p2) with respect to their native counterpart (a1–d1, e1–h1, i1–l1, and m1–p1). The original morphology of aortic valve cusps was well preserved after decellularization. Because of the high cellularity of both ventricles and interventricular septum, decellularization induced an important reduction of tissue thickness. Magnification bars: 200 μm for the aortic valve (a1–d1 and a2–d2)); 100 μm for the other tissues (e1–p1 and e2–p2).

After the decellularizing treatment based on 2,3-butanedione monoxime, protease inhibition, antioxidation, low SDS concentration for limited time, and nuclease digestion in perfusion/submersion conditions, rat hearts appeared macroscopically as white, translucent organ scaffolds with well-discernible internal components, as coronary arterial tree and interventricular/atrial septa. During treatment, in fact, progressive discoloration was achieved, suggestive of the loss of resident muscular cells (Figure 1A a and b).

As a first parameter of effective cell removal,^37^ residual DNA was quantified as 13.1 ± 5.8 ng/mg for decellularized organ scaffolds against 1145.8 ± 98.2 ng/mg for native hearts (Figure 1B). With a decrease of about 98.9% when compared to the native counterparts, the nucleic acid residue in decellularized organs was significantly reduced with respect to native tissues (*p* < 0.0001) and well below the threshold of 50 ng/mg per each decellularized sample, previously set by Crapo and colleagues^37^ as an indicator of effective cell extraction (Figure 1B).

In comparison to native tissues (Figure 1C a1–d1 for aortic valve, e1–h1 for right ventricle, i1–l1 for interventricular septum, and m1–p1 for left ventricle), histological analysis revealed effective cell removal in all organ structures, including heart valves, blood vessels, and ventricles with preservation of the original ECM histoarchitecture (Figure 1C a2–d2, e2–h2, i2–l2, and m2–p2). Cell extraction induced a substantial loss of wall thickness in both atria and ventricles and rendered appreciable the delicate and intricate ECM network (Figure 1C e2–h2, i2–l2, and m2–p2) that surrounds cardiac cells in native tissues (Figure 1C e1–h1, i1–l1, and m1–p1). In particular, the cardiovascular tree (blood vessels and heart valves), exploited to perfuse the heart and hence more exposed to the decellularizing solutions, appeared to be deprived of cell elements but maintained a conserved collagen/elastin scaffolding (Figure 1C a2–d2, e2–h2, and m2–p2), similarly to the typical ECM architecture observed in native samples for arteries and arterioles (Figure 1C a1–d1, e1–h1, and m1–p1). Generally, collagen, elastin, and mucopolysaccharides of decellularized hearts were likely unmodified in their distribution by comparison with native organs after histochemical staining (respectively, Figure 1C a2–d2, e2–h2, i2–l2, and m2–p2 versus a1–d1, e1–h1, i1–l1, and m1–p1).

### Steps 5–7: Cardiac ECM Was Not Altered in Its Composition and Architecture by the Decellularization Treatment

**Figure 2:**
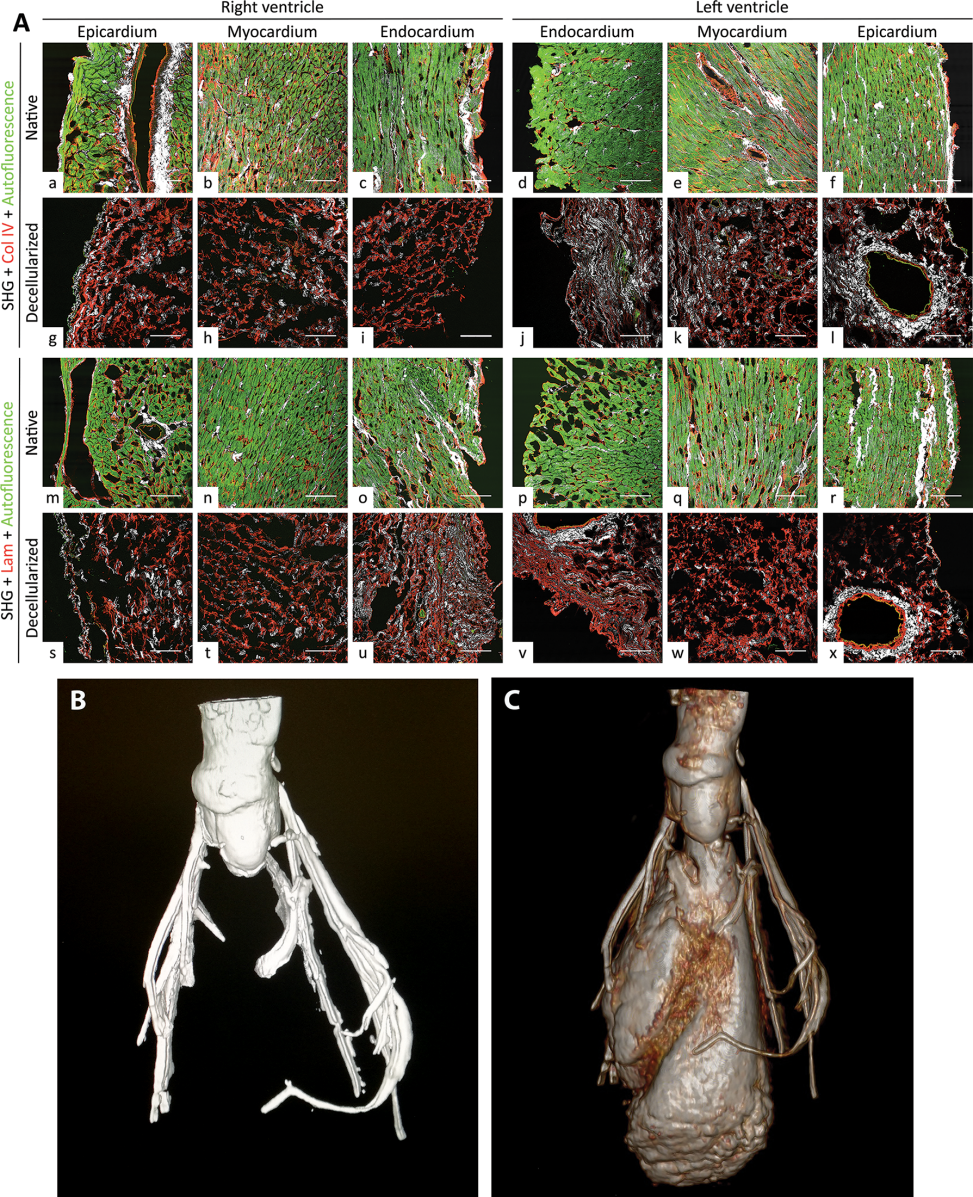
Evaluation of the preservation of basal membrane and coronary arterial tree of decellularized hearts. (A) The main constituents of basal membrane appeared intact in decellularized heart ventricles in two-photon tissue analysis. Collagen IV (Col IV) in red (for decellularized epicardium, myocardium, and endocardium, respectively; g, h, and i for right ventricle and l, k, and j for left ventricle, to be compared to native counterparts, i.e., a, b, and c for right ventricle, as well as f, e, and d for left ventricle). Laminin (Lam) in red (for decellularized epicardium, myocardium, and endocardium, respectively; s, t, and u for right ventricle and y, w, and x for left ventricle, to be compared to native counterparts, i.e., m, n, and o for right ventricle and p, q, and r for left ventricle). For each region of interest, collagen and elastin distributions are evidenced by second harmonic generation (SHG; in white) and autofluorescence (in green), respectively. Note the important reduction of the autofluorescence signal after decellularization. In native samples, it shows not only elastin but also resident cells of heart tissues. Magnification bars: 100 μm. (B) Microcomputed tomography of a decellularized heart. After injection of a contrast medium, the arterial coronary tree of decellularized hearts resulted intact and properly perfused without any leakage. (C) Three-dimensional reconstruction of a decellularized heart after microcomputed tomography analysis. In order to perform a ventriculography of each decellularized heart, the aortic valve was induced to its opened position, so that contrast medium could enter the internal chambers of the ventricles, too. The walls of the ventricles behaved as a barrier, blocking any leakage toward the external environment and indicating a well-preserved extracellular matrix.

The strong autofluorescence of cardiomyocytes and elastin observed in native hearts by TPM (Figure 2A a–f and m–r) was mostly lost after decellularization (Figure 2A g–l and s–x). In particular, only the elastic fibers contributed to this signal in decellularized hearts, whereas the sarcomere components, hallmarks of native cardiac tissues, were absent (Figure 2A g–l and s–x).

The SHG signal, mostly based on collagen I, was unmodified between decellularized and native hearts (Figure 2A, respectively, g–l and s–x versus a–f and m–r). After decellularization, basal lamina elements, as collagen IV (Figure 2A g–i for right ventricle and j–l for left ventricle) and laminin (Figure 2A s–u for right ventricle and v–x for left ventricle), also appeared well-preserved in epicardial, myocardial, and endocardial layers when compared to the native counterparts (respectively, Figure 2A a–c for right ventricle and d–f for left ventricle, and m–o for right ventricle and p–r for left ventricle). In particular, decellularized cardiac wall and blood vessels maintained the three-dimensional organization and distribution of the original basal membrane (Figure 2A, respectively g–l and s–x versus a–f and m–r).

ECM proteomic composition after decellularization confirmed the species origin of analyzed tissue scaffolds, i.e., *Rattus norvegicus*. Several collagen types, including I, IV, V, XV, and XVIII as well as procollagen I, were identified (Table 1). Other main components of cardiac ECM were revealed, such as fibronectin, an essential protein for cardiomyocytes and cardiac stem cells, and fibulin, an elastin precursor. Moreover, proteins fundamental for biological activity, e.g., for the basal membrane, as heparan sulfate, for extracellular matrix synthesis, as procollagens, for endothelial cell adhesion and myogenesis, and as keratins and dermatopontin, were evidenced (Table 1 and Table S1).

**Table 1:** Proteomic Analysis of Decellularized Hearts^a^

Accession	Protein ID	Score	# unique peptides	# peptides	# PSMs	MW (kDa)	Calc.d pI
F1LTJ5	heparan sulfate, Hspg2; OS = *Rattus norvegicus* GN = Hspg2 PE = 4 SV = 2 [F1LTJ5_RAT]E = 1	528.47	13	20	25	216.3	5.67
P02454	collagen alpha-1(I) chain; OS = *Rattus norvegicus* GN = Col1a1 PE = 1 SV = 5 [CO1A1_RAT]	372.44	8	8	9	137.9	5.92
F1M566	heparan sulfate, Hspg2 (fragment); OS = *Rattus norvegicus* GN = Hspg2 PE = 4 SV = 2 [F1M566_RAT]	345.11	3	10	11	230.7	6.95
F1MA59	collagen IV, Col4a1; OS = *Rattus norvegicus* GN = Col4a1 PE = 4 SV = 1 [F1MA59_RAT]	334.39	6	6	11	160.5	8.29
F1M6Q3	collagen IV, Col4a2; OS = *Rattus norvegicus* GN = Col4a2 PE = 4 SV = 2 [F1M6Q3_RAT]	165.72	6	6	6	166.1	8.65
F1LS40	collagen alpha-2(I) chain; OS = *Rattus norvegicus* GN = Col1a2 PE =4 SV = 2 [F1LS40_RAT]	219.09	6	6	8	129.8	9.22
D4A115	collagen VI, Col6a3; OS = *Rattus norvegicus* GN = Col6a3 PE = 4 SV = 2 [D4A115_RAT]	161.83	2	2	3	240.0	6.38
F1LPD0	collagen XV, Col15a1 (fragment); OS = *Rattus norvegicus* GN = Col15a1 PE = 1 SV = 2 [F1LPD0_RAT]	149.06	5	5	5	137.0	4.91
F1M0B2	protein LOC683295 (fragment); OS = *Rattus norvegicus* GN = LOC683295 PE = 4 SV = 2 [F1M0B2_RAT]	144.17	2	2	2	57.7	7.88
P02770	serum albumin; OS = *Rattus norvegicus* GN = Alb PE = 1 SV =2 [ALBU_RAT]	137.08	5	5	7	68.7	6.48
G3 V763	collagen alpha-1(V) chain; OS = *Rattus norvegicus* GN = Col5a1 PE = 4 SV = 2 [G3 V763_RAT]	135.61	2	2	3	161.7	4.83
Q6IMF3	keratin, type II cytoskeletal 1; OS = *Rattus norvegicus* GN = Krt1 PE = 2 SV = 1 [K2C1_RAT]	114.80	3	3	3	64.8	7.87
Q6IFW6	keratin, type I cytoskeletal 10; OS = *Rattus norvegicus* GN = Krt10 PE = 3 SV = 1[K1C10_RAT]	116.13	5	5	5	56.5	5.15
F1M6Q3	protein Col4a2; OS = *Rattus norvegicus* GN = Col4a2 PE = 4 SV = 2 [F1M6Q3_RAT]	95.31	3	3	4	166.1	8.65
Q9WVH8	fibulin-5; OS = *Rattus norvegicus* GN = Fbln5 PE = 2 SV = 1 [FBLN5_RAT]	86.76	2	2	3	50.1	4.67
F1LST1	fibronectin; OS = *Rattus norvegicus* GN = Fn1 PE = 4 SV =2 [F1LST1_RAT]	85.80	3	3	4	202.4	539
F1LR02	procollagen, type XVIII, alpha 1, isoform CRA_a; OS = *Rattus norvegicus* GN = Col18a1 PE = 4 SV = 2 [F1LR02_RAT]	69.29	2	2	2	134.6	6.29
F1LNH3	procollagen, type VI, alpha 2, isoform CRA_a; OS = *Rattus norvegicus* GN = Col6a2 PE =4 SV = 2 [F1LNH3_RAT]	34.71	2	2	2	109.6	6.61
D4A9H2	dermatopontin (predicted), isoform CRA_c; OS = *Rattus norvegicus* GN = Dpt PE = 4 SV = 1 [D4A9H2_RAT]	54.82	2	2	2	20.0	5.15

a Legend: Protein ID, protein identifier; score: relative abundance of the protein in tested sample; PSM: peptide-spectrum matching; MW, molecular weight; Calc.d pI, calculated isoelectric point.

In addition, the coronary arterial tree appeared to be preserved after decellularization, as observed by microcomputed tomography analyses and three-dimensional reconstruction (Figure 2B, C). To better assess the competence of decellularized heart scaffolds after evaluation of the coronary tree, we induced the aortic valve to the open position to allow the contrast medium filling into the ventricular chambers and perform heart ventriculography. Leakage of contrast medium from the coronary arteries (Figure 2B, C) or from the walls (Figure 2C) was absent at any evaluation stage of loaded decellularized hearts.

### Step 8: Cytocompatibility of Heart ECM Scaffolds Was Maintained after Decellularization Treatment

**Figure 3:**
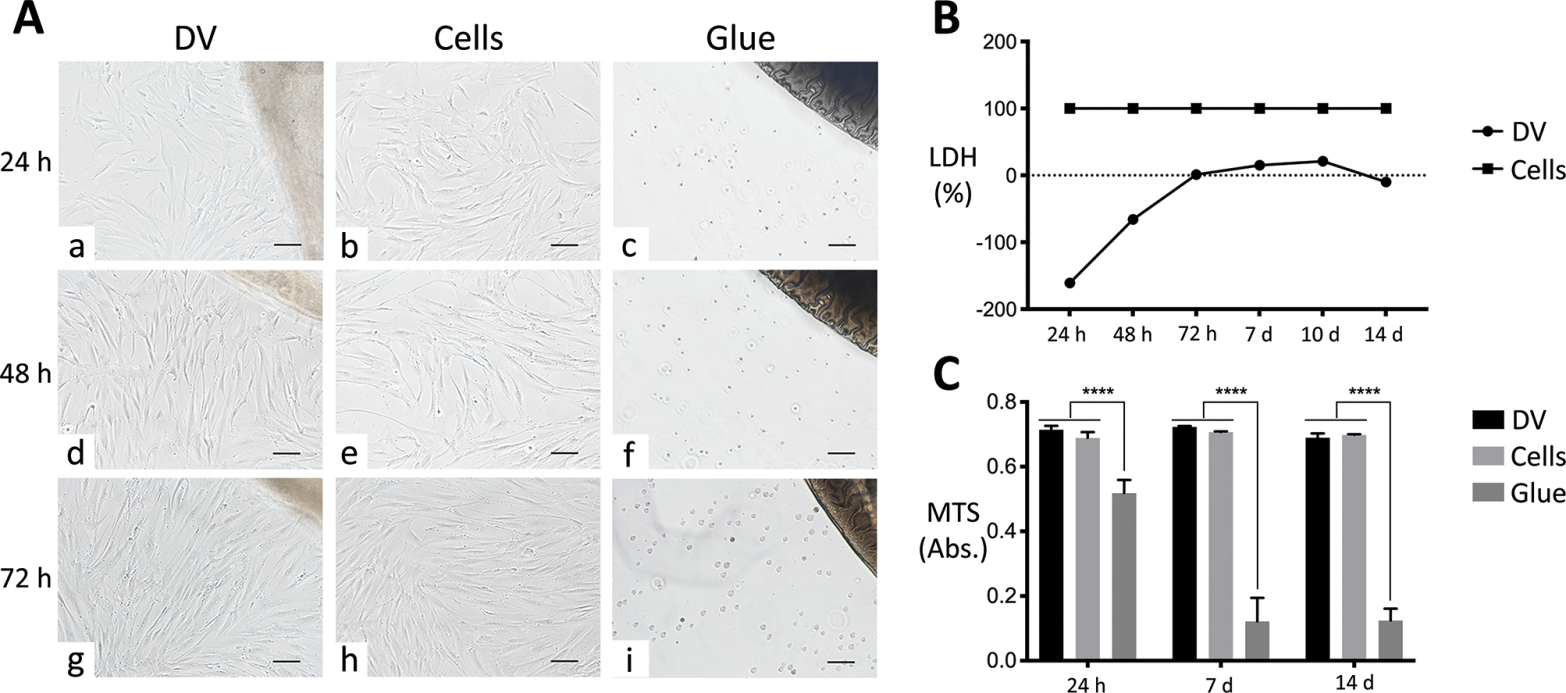
Cytocompatibility evaluation of decellularized heart ventricles. (A) Direct contact assay of decellularized ventricles (DVs) with human bone marrow mesenchymal stem cells (indicated as Cells). Phenotype and number of cells in contact with DVs (a, d, and g) were similar to those cultivated directly on plastics (b, e, and h) at all time points considered (24, 48, and 72 h). In direct contact with DVs, cells were shown to progressively polarize toward the decellularized tissues (a, d, and g). In the presence of glue, mesenchymal stem cells showed critical signs of sufferance and death (c, f, and i), immediately losing any morphological hallmarks. The DV scaffolds are visible in the upper right corner of a, d, and g, whereas the glue spots can be seen in the same position of c, f, and i. Magnification bars: 100 μm. (B) Cell cytotoxicity. Lactate dehydrogenase (LDH) activity in mesenchymal stem cells in direct contact with DVs normalized to those in culture plastics was negligible for all time points considered (24, 48, and 72 h, as well as 7, 10, and 14 days), differently from the same cells in the presence of glue, for which the value was 100%. (B) Cell viability. At each time point (24 h and 7 and 14 days), no significant alteration in the proliferation activity of mesenchymal stem cells was observed after contact with DVs with respect to the plastic culture support, as measured with MTS colorimetric assay. Conversely, glue significantly reduced cell proliferation with respect to the other culture conditions. Data are expressed as mean ± standard deviation (****, *p* < 0.0001).

At each considered time (24, 48, and 72 h), no modifications in cell morphology were observed for hBM-MSCs in direct contact with decellularized rat heart ventricles with respect to cells growing onto traditional plastic support (Figure 3A a, d and g versus b, e and h). In particular, no signs of stress, as intracytoplasmic vacuoles or stress fibers, and unaltered doubling time were revealed. A polarized cell growth toward decellularized scaffolds was evidenced over time (Figure 3A a, d and g). Conversely, cells cultured in the presence of cyanoacrylate glue appeared to be suffering, as they did not display the typical morphological pattern and were unable to adhere and/or survive (Figure 3A c, f, and i).

The percentage of cytotoxicity induced by acellular ventricle scaffolds on hBM-MSCs was close to zero at all considered time points, slightly rising around day 10 and then progressively decreasing after 2 weeks. Because the glue was considered as the most cytotoxic condition (100%, grade 5 according to ISO 10993-5 classification^35^), the normalized LDH activity in the heterologous interaction between decellularized rat scaffolds and hBM-MSCs was so low that it reached negative percentage values (grade 0^35^) (Figure 3B).

**Figure 4:**
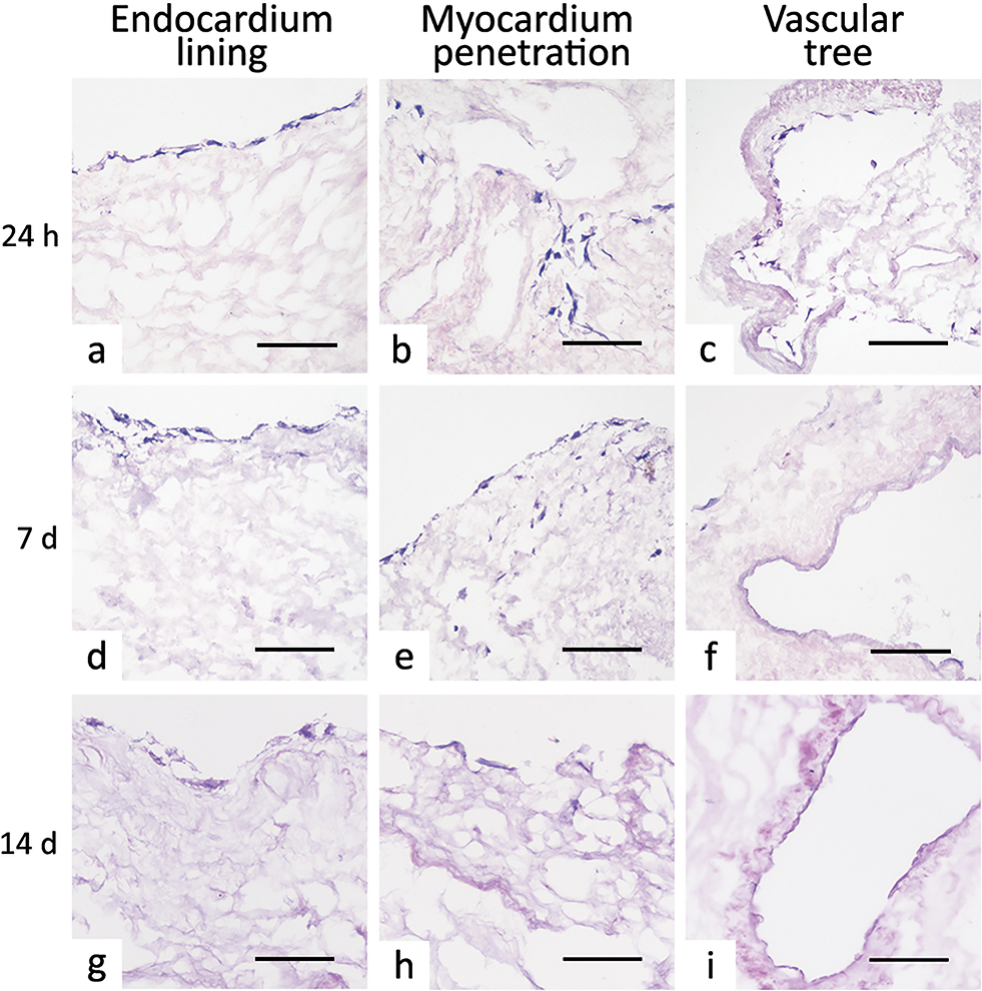
Cell homing behavior of decellularized ventricle scaffolds. Already after 24 h, human bone marrow mesenchymal stem cells seeded onto rat decellularized ventricles formed a complete-like lining on the endocardial surface (a at 24 h, d at 7 days, and g at 14 days) and could be observed penetrating in the myocardial layer (b at 24 h, e at 7 days, and h at 14 days) and covering the intimal scaffolding of the vascular tree, i.e., blood vessels and heart valve apparatus (c at 24 h, f at 7 days, and i at 14 days). Magnification bars: 100 μm.

Proliferation activity of hBM-MSCs seeded onto decellularized ventricles and onto polystyrene remained unaltered over time without variations in between the two groups. Conversely, it was statistically different with respect to the highly cytotoxic glue culturing condition (Figure 3C, *p* < 0.0001). Already at 24 h from the static seeding, human cells appeared to have adhered to the xenogeneic decellularized scaffolds, by forming a continuous-like monolayer, lining their endocardial surface at the following considered time points, too (Figure 4 a, d, and g at 24 h and 7 and 14 days, respectively). In-depth penetration in the myocardial layers was observed more importantly at 7 and 14 days (Figure 4 b, e, and h at 24 h and at 7 and 14 days, respectively), with spread into the perivascular space of the coronary tree (Figure 4 c at 24 h, f at 7 days, and i at 14 days).

## Discussion

An effective decellularization method for the heart should represent an optimal compromise between cell removal and preservation of the ECM proteins for all of its different structures (basal membrane of endocardium and myocardium, heart valves, coronary arterial tree, etc.). This outcome has to be reached through a conservative decellularization approach, conceived to reduce any side variation hampering the bioactivity of the ECM. In particular, the applied decellularization treatment should not leave residues of the agents used, which could be able to induce cytotoxicity and, thus, hinder cell repopulation of the scaffold. Moreover, the time factor in the preparation of a whole bioengineered heart, comprising decellularization and repopulation, is particularly relevant in the perspective of an urgent need to treat a cardiopathic patient with a failing heart.

After the first heart decellularization performed by Ott et al., many other methods have been developed to obtain a whole cardiac scaffold from several mammalian organs.^15^ These extraction treatments generally exploit coronary arterial tree perfusion to circulate the whole organ with solutions based on detergents, chemical agents, and/or enzymes, possibly combined with physical conditioning.

In this study, an optimized decellularization method for conservative heart decellularization has been proposed by applying a low concentration of detergents for a short time and facilitating cell extraction by means of myorelaxation and osmotic shock in antioxidant and nonenzymatic denaturing conditions.

With respect to other published methods that we recently reviewed,^15^ this decellularization shares the retrograde perfusion through the aorta and the use of SDS and Triton X100, as detergents. However, this approach is realized by combining submersion with flow-controlled perfusion concurrently and making use of half the concentration of SDS generally utilized. This ionic detergent is well-known to be a denaturing agent at a concentration equal or superior to 1%, by inducing collagen and elastin precipitation, as well as strongly affecting glycosaminoglycans moiety.^38^ Therefore, a decellularization protocol based on a low concentration of SDS is more conservative. Also, Momtahan et al. applied 0.5% SDS perfusion in their decellularization protocol to frozen hearts (in this case, porcine ones).^20^ In our experience, we observed that the use of freeze–thaw cycles may facilitate the tissue/organ devitalization by inducing a physical detachment of cells from the ECM. However, it can be very detrimental to the latter, even when controlled cryopreservation and thawing are performed, as also seminally evidenced by the literature on the topic.^39−41^

With the aim of better preserving cardiac ECM during decellularization, we designed our protocol by excluding enzymatic treatments able to induce denaturation. In other heart decellularization protocols,^18,42,43^ the enzyme trypsin has been applied in combination with chelating agents and detergents at a varying concentration between 0.05 and 0.2%. This serine protease is very effective in cell detachment, as commonly applied in cell expansion procedures. Its optimal activity is exercised during specific environmental conditions, such as 37 °C temperature and short-time effectiveness. As such, a continuous solution refreshment and long incubations should be required to treat a high cellular density organ, like the heart. A further strong disadvantage of the use of trypsin in tissue and organ decellularization is given by its aggressiveness toward the ECM. Collagen and elastin may undergo chemical modifications that are able to compromise their biomechanical and bioactive properties.^37^ The low resistance to trypsinization exhibited by collagens could be particularly detrimental for the delicate and thin cardiomyocyte basal membranes, mainly composed of collagen IV. In the independent studies by Akhyari et al. and Merna et al.,^18,43^ heart decellularization protocols using trypsin and/or detergents were compared in terms of efficacy. Both concluded that decellularization could be achieved with trypsin/detergent combination, but less effectively than with sole detergent cocktails in terms of cell removal and ECM preservation. In addition, when trypsin was applied alone to decellularize hearts,^43^ generated scaffolds were only partially acellular and severely deteriorated in their original biomechanical behavior. In the balance between benefits and shortcomings related to its application in the decellularization of the heart, trypsin cannot be considered as an ideal decellularizing agent either when coupled with detergents in strictly controlled experimental conditions or alone.

Besides the absence of denaturing enzymatic agents, we introduced a further preservation strategy in our protocol by performing a pretreatment based on a cocktail of protease inhibitors. As we demonstrated previously,^10−12,27^ the inhibition of metalloproteinases released by lysed cells is essential to maintain the integrity of the tissue ECM during decellularization.

Another particular characteristic of the hereby applied decellularization method is the exposure time to SDS, which has been importantly reduced to 5.5 h, against 12 h applied in other protocols.^15^ The reduction of the incubation time with SDS has been rendered feasible by a preincubation of the cardiac organs in conditions favoring cell relaxation (2,3-butanedione monoxime) and oxidation protection. Through this dual action strategy, the detergent activity is facilitated and, even at a very low concentration, an incubation time reduced by more than double is sufficient to obtain a successful decellularization yield, potentiated by a final step to remove all nucleic acids (Benzonase).^10−12,27^ Indeed, the effective removal of cells and especially DNA is an important aspect for the future biocompatible clinical use of decellularized scaffolds since these cell remains may elicit inflammatory response and immune reactions,^44^ as well as calcification^10,11^ once in vivo. Although the reduced detergent concentration and incubation time, this protocol met the indications suggested in the literature by Crapo and colleagues^37^ and achieved a higher, if not comparable, DNA reduction (i.e., nearly 99%) with respect to other detergent-based methods applied to the whole rat heart. Ott and colleagues^9^ obtained removal of around 96% of the native DNA, whereas Akhyari et al.^18^ observed a reduction of more than 99% by using protocols based on the combination of SDS, deoxycholate, and DNase.

This carefully designed protocol applied for heart decellularization brought about a very well preserved cardiac ECM. The composition and three-dimensional distribution of its elements are, in fact, maintained in all cardiac wall layers, heart valve apparatus (semilunar cusps, atrioventricular leaflets, and sinuses of Valsalva), and blood vessels (aorta, coronary arteries, arterioles, veins, etc.), as confirmed by histological, combined immunofluorescence-TPM, micro-CT, and proteomic characterization. More specifically, vascular patency and valve competence are indispensable functions to guarantee the pump functionality of the heart and consequently, the unidirectionality of blood flow in the body. Although a retrograde perfusion of the ascending aorta through its cannulation was used to decellularize the cardiac organ, no deleterious effects on the mechanical characteristics of blood vessels and heart valves were evidenced by micro-CT. This observation is in line with previous reports by other groups utilizing different decellularizing techniques.^18,45^

The coronary vascular tree will also be exploited in the repopulation phase; therefore, the maintenance of its integrity is fundamental to properly reconstructing the specialized parenchyma of the heart. The preservation of collagen IV and laminin observed after decellularization is a promising sign for a successful re-endothelialization of the blood-contacting surfaces, thus possibly preventing thrombotic events and enabling cell migration.^10,11^ With reference to the muscle-rich tissues of the heart, the preservation of the 3D spatially organized basal membrane should offer a friendly environment for cardiomyocytes, notoriously very delicate and unstable cells. Moreover, the maintenance of collagen XV and XVIII is favorable considering the endothelial cell adhesion and migration, as well as cardiomyocyte mechanical stability, which are guaranteed in native conditions by these nonfibril-forming ECM proteins. These nonfibrillar collagens highly interact with heparan sulfate, fibronectin, and alpha1, beta1 integrin,^46,47^ essential for many functions of the cardiovascular cells. All of these proteins were evidenced to be preserved in the scaffolds by the proteomic analysis. The pivotal role of these proteins has also been evidenced in the biology and physiology of the cardiac stem cell niches, namely, the endogenous reservoir of the tissue regeneration power in the heart.^48−50^ Other proteins shown by the analysis of the full proteome of decellularized hearts, such as collagen V, dermatopontin, and fibulin, are also key players in novel ECM generation by exerting pro-synthetic, proliferative, pro-migratory, and pro-differentiating effects on surrounding cells. As such, the heart organ scaffolds decellularized with this new approach possess all the premises of an unaffected original biocompatibility.

Before testing the interaction with the typical differentiated parenchymal cells of the heart, we evaluated whether the bioactivity of decellularized cardiac scaffolds of rat origin was actually preserved by challenge with a human line of immature cells, as mesenchymal stem cells derived from a human healthy subject, by following the guidelines of ISO 10993-5.^35^ Although some species-specificity exists, most of the domains of ECM proteins are highly conserved in mammals. Therefore, the trivial cytotoxicity and optimal proliferation index observed for hBM-MSCs in contact with rat decellularized ventricles (heterologous interaction) can be considered as hallmarks of unaltered biocompatibility of these tissue/organ scaffolds after the decellularization treatment applied, as previously documented by Sánchez and co-workers.^45^ The constitution of a continuous-like lining onto the acellular endocardial surface, the progressive penetration in the myocardium basal membrane layer, and the proneness of hBM-MSCs to cover the intimal scaffolding of the vascular tree and valve apparatus confirm the homing microenvironment possessed by obtained cardiac ECMs. Ongoing experiments are verifying whether more differentiated cell lines distinctive of the heart organ, such as, for instance, endothelial cells and cardiomyocytes, derived from the cardiovascular commitment of human induced pluripotent stem cells, might adhere, proliferate, and/or spread onto/into decellularized ventricular scaffolds, before attempting the repopulation of the whole organ.

Because of the small size of each acellular rat heart, we could not perform any quantification of residual detergent on the relatively trivial amount of obtained scaffold by following the modality used for other decellularized cardiovascular tissues.^12^ However, we are confident that the positive outcomes observed in the cytocompatibility experiments might be indirectly suggestive of an efficient removal of the cytotoxic SDS by the action of Triton X100 and final wash out in PBS.

Relatively low experimental material due to the animal organ size, as well as experimental conditions applied for two-photon microscopic and proteomic studies may have represented some disadvantages in this study toward a fully quantitative assessment. Although statistical considerations are not complete for all the analyses performed, the univocal observations obtained with different, independent techniques in terms of effective decellularization and ECM preservation provide assurance of the real efficacy of the novel decellularization method we developed for the whole heart organ.

With the perspective of upscaling to human-size organs for translation into clinics, this approach might present another limitation. The success of any decellularization protocol is profoundly affected by many factors, with tissue/organ size as one of the most influencing. Therefore, although very promising with respect to the rat organ (nearly 1.5 cm × 1 cm diameter, about 1–2 g), the method applied hereby needs to be adjusted for the successful decellularization of larger hearts (nearly 12–13 cm × 8–9 cm diameter, about 230–350 g).

Ongoing research is aimed at adapting the current protocol to larger mammalian hearts with a suitable size for clinical translation, such as the porcine and human ones.

## Conclusions

Acellular, original cardiac scaffolds with well-preserved ECM integrity and unmodified bioactivity can be obtained by perfusion/submersion-based decellularization of whole hearts through a limited time exposure to a very low SDS concentration by means of protease inhibition and excitation–contraction cell uncoupling. Although scaling up to human-like organs is necessary for clinical translation, these preliminary results are supportive for the generation of a cardiac scaffold endowed with all the native ECM properties, thus acting as an informed footprint for functional heart regeneration.

## Supporting Information Available

Table S1, complete protein data set identified by proteomic analysis (PDF)

## Dedication

This study is dedicated to the memory of Michele Bellucci (1988–2018), a dear friend and young biotechnology scientist, who, affected by heart failure, lived with incredible enthusiasm and passion for science and regenerative medicine.

## Abbreviations

BCA: bicinchoninic acid
calc.d pI: calculated isoelectric point
ECM: extracellular matrix
FDR: false discovery rate
DVs: decellularized ventricles
hBM-MSCs: human bone marrow mesenchymal stem cells
HPLC: high performance liquid chromatography
LC: liquid chromatography
LDH: lactate dehydrogenase
LTQ: linear trap quadrupole
micro-CT: micro computed tomography
MS: mass spectrometry
MW: molecular weight
PBS: phosphate buffered saline
Protein ID: protein identifier
PSM: peptide-spectrum matching
RT: room temperature
SHG: second harmonic generation
SDS: sodium dodecyl sulfate
TPM: two-photon microscope
