# Supplementary material for: RegenHeart: A Time-Effective, Low-Concentration, Detergent-Based Method Aiming for Conservative Decellularization of the Whole Heart Organ
Source: ACS Biomater Sci Eng. 2020 Aug 20;6(10):5493–506. doi: 10.1021/acsbiomaterials.0c00540 (PMC8011801; doi:10.1021/acsbiomaterials.0c00540)
Supplement: Supplementary file 1 — ab0c00540_si_001.pdf [file ab0c00540_si_001.pdf]

RegenHeart: a time-effective, low-concentration detergent-based method aiming for conservative decellularization of the whole heart organ

Eleonora Dal Sasso[1, ‡], Roberta Menabò[2, 3, ‡], Davide Agrillo[1], Giorgio Arrigoni[3], Cinzia Franchin[3], Chiara Giraudo[4,5], Andrea Filippi[6,7,8], Giulia Borile[6,8], Guido Ascione[1], Fabio Zanella[9], Assunta Fabozzo[5,9], Raffaella Motta[4], Filippo Romanato[5,6,8], Di Lisa Fabio[2,3], Laura Iop[1,5,+,*], and Gino Gerosa[1,5,9, +]

[1] Cardiovascular Regenerative Medicine, Department of Cardiac Thoracic Vascular Sciences and Public Health, University of Padua, Padua, Italy;

[2] Institute of Neuroscience, National Research Council (CNR), Padua, Italy;

[3] Department of Biomedical Sciences, University of Padua, Padua, Italy

[4] Department of Medicine, University of Padua, Padua, Italy

[5] L.I.F.E.L.A.B. Program, Consorzio per la Ricerca sanitaria (CORIS), Veneto Region, Padua, Italy;

[6] Department of Physics and Astronomy ‘G. Galilei’, University of Padua, Padua, Italy.

[7] Fondazione Bruno Kessler, Trento, Italy.

[8] Institute of Pediatric Research ‘Città della Speranza’, Padua, Italy

[9] Cardiac Surgery Unit, University Hospital of Padua, Padua, Italy

Number of pages: 11

Number of tables: 1 (Table S1)

Table S1: Complete protein dataset identified by proteomic analysis

Accession	Description	Score	Coverage	# Proteins	# Unique Peptides	# Peptides	# PSMs	# AAs	MW [kDa]	calc. pI
F1LTJ5	Protein Hspg2 OS=Rattus norvegicus GN=Hspg2 PE=4 SV=2 - [F1LTJ5_RAT]	742,73	19,24	1	19	27	35	2001	216,3	5,67

A2	Sequence	# PSMs	# Proteins	# Protein Groups	Protein Group Accessions	Modifications	ΔCn	q-Value	PEP	IonScore	Exp Value	Charge	MH+ [Da]	ΔM [ppm]	RT [min]	# Missed Cleavages
High	SPAYTLVWTR	1	2	2	F1M566;F1LTJ5		0,0000	0	0,06	78	1,48E-07	2	1193,63176	0,33	28,02	0
High	EVSEAVVEKLETEYVK	2	1	1	F1LTJ5		0,0000	0	0,12	64	3,32E-06	2	1851,95720	-0,80	29,95	1
High	LPAIEPSDQGQYLcR	2	2	2	F1M566;F1LTJ5	C14(Carbamidomethyl)	0,0000	0	0,08	63	4,86E-06	2	1746,85063	1,50	25,59	0
High	SIEYSPQLEDANAK	1	1	1	F1LTJ5		0,0000	0	0,14	61	6,23E-06	2	1564,74944	0,28	21,53	0
High	AEmLQALASLEAVLLQTVYNTK	1	1	1	F1LTJ5	M3(Oxidation)	0,0000	0	0,1	59	6,11E-06	3	2422,29032	0,17	47,14	0
High	AELLVAEAPSKPITVTVEEQR	1	2	2	F1M566;F1LTJ5		0,0000	0	0,03	54	1,68E-05	3	2280,24479	0,05	27,70	0
High	mTSVGLSDIVmDTTVTYTTIHGR	1	1	1	F1LTJ5	M1(Oxidation); M11(Oxidation)	0,0000	0	0,09	53	3,69E-05	3	2530,21909	1,03	32,92	0
High	GmVFGIPDGVLELVPQR	3	1	1	F1LTJ5	M2(Oxidation)	0,0000	0	0,07	51	8,06E-05	2	1842,98015	0,98	38,34	0
High	LSGISmDVAVPENTGQDPAR	1	1	1	F1LTJ5	M6(Oxidation)	0,0000	0	0,15	48	0,000118	2	2072,99394	1,02	23,92	0
High	IPGDQIVSVVFIK	2	1	1	F1LTJ5		0,0000	0	0,15	47	6,32E-05	3	1414,82993	-0,40	34,52	0
High	LNFDQPSDFK	1	1	1	F1LTJ5		0,0000	0	0,25	43	0,000348	2	1210,57500	0,88	24,66	0
High	ScNEcSEGSFHLSK	1	1	1	F1LTJ5	C2(Carbamidomethyl); C5(Carbamidomethyl)	0,0000	0	0,22	39	0,000119	3	1641,66281	-0,26	17,63	0

	High	AAGVPSASITWR	1	2	2	F1M566;F1LTJ5		0,0000	0	0,25	38	0,002495	2	1215,64824	0,11	24,52	0
	High	RFLVHDAFWALPK	1	1	1	F1LTJ5		0,0000	0	0,22	38	0,000904	3	1599,87979	0,17	29,02	1
	High	FLVHDAFWALPK	1	1	1	F1LTJ5		0,0000	0	0,21	37	0,001983	3	1443,77781	-0,41	32,32	0
	High	ALSSAGQHVVR	1	2	2	F1M566;F1LTJ5		0,0000	0	0,39	36	0,001487	3	1124,61811	0,87	14,59	0
	High	SQSVRPGADVTFIcTAK	1	2	2	F1M566;F1LTJ5	C14(Carbamidomethyl)	0,0000	0	0,13	36	0,002418	3	1836,92539	-1,07	22,50	0
	High	LETEYVK	1	1	1	F1LTJ5		0,0000	0	0,35	36	0,002108	2	881,46239	1,01	17,28	0
	High	GHTPTHPGALNQR	2	1	1	F1LTJ5		0,0000	0	0,42	36	0,002974	3	1385,70179	-1,11	12,74	0
	High	ATATAcRPcPcPYIDASQR	1	1	1	F1LTJ5	C6(Carbamidomethyl); C9(Carbamidomethyl); C11(Carbamidomethyl)	0,0000	0	0,21	32	0,002308	3	2194,97916	0,01	19,58	0
	High	GmLEPVQKPDVILVGAGYR	1	1	1	F1LTJ5	M2(Oxidation)	0,0000	0	0,14	31	0,005688	3	2058,10654	0,59	27,51	0
	High	VASGLYLGTcER	1	1	1	F1LTJ5	C10(Carbamidomethyl)	0,0000	0	0,18	29	0,012383	2	1325,65105	-0,62	21,66	0
	High	AmLQVHGGSGPR	1	2	2	F1M566;F1LTJ5	M2(Oxidation)	0,0000	0	0,24	29	0,013767	3	1225,61130	0,50	14,13	0
	High	VTSYGGELR	1	1	1	F1LTJ5		0,0000	0	0,43	29	0,016641	2	981,50163	1,64	17,35	0
	High	QLISTHFAPGDFQGFALVNPQR	3	1	1	F1LTJ5		0,0000	0	0,23	24	0,02766	3	2443,25241	0,25	34,39	0
	High	SVVPQGGPHSLR	1	2	2	F1M566;F1LTJ5		0,0000	0	0,43	23	0,042849	3	1233,67096	0,86	17,23	0
	High	QVQLSEEHWVHESGRPVQR	1	1	1	F1LTJ5		0,0000	0	0,3	12	0,462705	4	2301,14809	-0,13	19,65	0
P02454	Collagen alpha-1(I) chain OS=Rattus norvegicus GN=Col1a1 PE=1 SV=5 - [CO1A1_RAT]	402,81	6,81	1	8	8	11	1453	137,9	5,92							
	A2	Sequence	# PSMs	# Proteins	# Protein Groups	Protein Group Accessions	Modifications	ΔCn	q-Value	PEP	IonScore	Exp Value	Charge	MH+ [Da]	ΔM [ppm]	RT [min]	# Missed Cleavages
	High	GETGPAGPAGPIGPAGAR	1	1	1	P02454		0,0000	0	0,02	90	9,06E-09	2	1532,78325	1,02	19,70	0

	High	NGDRGETGPAGPAGPIGPAGAR	2	1	1	P02454		0,0000	0	0,03	72	5,44E-07	2	1974,97661	1,24	18,26	1
	High	GETGPSGPAGPTGAR	1	1	1	P02454		0,0000	0	0,06	70	9,24E-07	2	1311,63030	1,10	14,90	0
	High	DGEAGAQGAPGPAGPAGER	1	1	1	P02454		0,0000	0	0,05	68	1,01E-06	2	1664,76299	0,34	16,56	0
	High	SAGVSVPGPmGPSGPR	2	1	1	P02454	M10 (Oxidation)	0,0000	0	0,12	56	1,73E-05	3	1468,72089	-0,34	19,67	0
	High	GPAGPQGPR	2	1	1	P02454		0,0000	0	0,14	46	0,000201	2	836,43791	0,64	12,83	0
	High	GSEGPQGVR	1	1	1	P02454		0,0000	0	0,38	39	0,00089	2	886,43779	0,02	13,24	0
	High	GFSGLDGAK	1	1	1	P02454		0,0000	0	0,26	38	0,001526	2	851,42571	-0,12	19,23	0
F1M566	Protein Hspg2 (Fragment) OS=Rattus norvegicus GN=Hspg2 PE=4 SV=2 - [F1M566_RAT]	397,78	9,65	1	6	14	16	2165	230,7	6,95							
	A2	Sequence	# PSMs	# Proteins	# Protein Groups	Protein Group Accessions	Modifications	ΔCn	q-Value	PEP	IonScore	Exp Value	Charge	MH+ [Da]	ΔM [ppm]	RT [min]	# Missed Cleavages
	High	SPAYTLVWTR	1	2	2	F1M566;F1LTJ5		0,0000	0	0,06	78	1,48E-07	2	1193,63176	0,33	28,02	0
	High	ITVPASEGSSYR	1	1	1	F1M566		0,0000	0	0,13	66	2,45E-06	2	1266,63323	0,57	19,19	0
	High	LPAIEPSDQGQYLcR	2	2	2	F1M566;F1LTJ5	C14(Carbamidomethyl)	0,0000	0	0,08	63	4,86E-06	2	1746,85063	1,50	25,59	0
	High	AELLVAEAPSKPITVTVEEQR	1	2	2	F1M566;F1LTJ5		0,0000	0	0,03	54	1,68E-05	3	2280,24479	0,05	27,70	0
	High	SGIVQSGSVIR	1	1	1	F1M566		0,0000	0	0,15	54	4,2E-05	2	1102,62175	0,18	19,63	0
	High	IAHVELADAGQYR	1	1	1	F1M566		0,0000	0	0,18	44	0,000403	3	1442,74033	1,12	21,00	0
	High	SPVISIEPPSSTVQQGQDASFK	1	1	1	F1M566		0,0000	0	0,14	43	0,000447	3	2302,15628	0,02	27,99	0
	High	AAGVPSASITWR	1	2	2	F1M566;F1LTJ5		0,0000	0	0,25	38	0,002495	2	1215,64824	0,11	24,52	0
	High	ALSSAGQHVVR	1	2	2	F1M566;F1LTJ5		0,0000	0	0,39	36	0,001487	3	1124,61811	0,87	14,59	0

	High	SQSVRPGADVTFIcTAK	1	2	2	F1M566;F1LTJ5	C14(Carbamidomethyl)	0,0000	0	0,13	36	0,002418	3	1836,92539	-1,07	22,50	0
	High	IASVKPSDAGTYVcQAQNALGTAQK	1	1	1	F1M566	C14(Carbamidomethyl)	0,0000	0	0,12	33	0,003517	3	2578,29398	0,35	27,83	0
	High	AmLQVHGGSGPR	1	2	2	F1M566;F1LTJ5	M2(Oxidation)	0,0000	0	0,24	29	0,013767	3	1225,61130	0,50	14,13	0
	High	SVVPQGGPHSLR	1	2	2	F1M566;F1LTJ5		0,0000	0	0,43	23	0,042849	3	1233,67096	0,86	17,23	0
	High	GGSLPAGHQVHGHILR	2	1	1	F1M566		0,0000	0	0,3	15	0,234476	4	1635,88369	0,60	15,77	0
F1MA59	Protein Col4a1 OS=Rattus norvegicus GN=Col4a1 PE=4 SV=1 - [F1MA59_RAT]	334,39	5,81	1	6	6	11	1669	160,5	8,29							
	A2	Sequence	# PSMs	# Proteins	# Protein Groups	Protein Group Accessions	Modifications	ΔCn	q-Value	PEP	IonScore	Exp Value	Charge	MH+ [Da]	ΔM [ppm]	RT [min]	# Missed Cleavages
	High	HSQTTDDPLcPPGTK	2	1	1	F1MA59	C10(Carbamidomethyl)	0,0000	0	0,02	72	3,41E-07	2	1653,75505	0,77	17,35	0
	High	FSTmPFLFcNINNVcNFASR	4	1	1	F1MA59	M4(Oxidation); C9(Carbamidomethyl); C15(Carbamidomethyl)	0,0000	0	0,07	71	3,17E-07	2	2455,10771	3,44	36,06	0
	High	AHGQDLGTAGScLR	2	1	1	F1MA59	C12(Carbamidomethyl)	0,0000	0	0,07	55	1,89E-05	2	1442,68108	0,36	16,47	0
	High	NDYSYWLSTPEPmPmSmAPISGDNIRPFISR	1	1	1	F1MA59	M13(Oxidation); M15(Oxidation); M17(Oxidation)	0,0000	0	0,4	52	1,81E-05	3	3620,64951	-0,03	33,43	0
	High	KFSTmPFLFcNINNVcNFASR	1	1	1	F1MA59	M5(Oxidation); C10(Carbamidomethyl); C16(Carbamidomethyl)	0,0000	0	0,44	44	0,000183	3	2583,19846	1,63	33,44	1
	High	ILYHGYSLLYVQGNER	1	1	1	F1MA59		0,0000	0	0,2	38	0,001474	3	1924,99179	0,10	27,15	0

F1LS40	Collagen alpha-2(I) chain OS=Rattus norvegicus GN=Col1a2 PE=4 SV=2 - [F1LS40_RAT]	246,08	4,52	2	6	6	7	1372	129,8	9,22						
	A2	Sequence	# PSMs	# Proteins	# Protein Groups	Protein Group Accessions	Modifications	ΔCn	q-Value	PEP	IonScore	Exp Value	Charge	MH+ [Da]	ΔM [ppm]	RT [min]	# Missed Cleavages
	High	GVSAGPGPmGLmGPR	1	2	1	F1LS40	M9(Oxidation); M12(Oxidation)	0,0000	0	0,05	84	3,2E-08	2	1415,67815	0,75	19,04	0
	High	GEAGAAGPSGPAGPR	1	2	1	F1LS40		0,0000	0	0,04	68	1,67E-06	2	1251,60967	1,55	14,58	0
	High	GPAGPSGPIGK	1	2	1	F1LS40		0,0000	0	0,09	53	3,66E-05	2	937,50963	-0,63	15,44	0
	High	GPAGPSGPIGKDGR	1	2	1	F1LS40		0,0000	0	0,12	52	7,88E-05	2	1265,66069	0,74	14,86	1
	High	GPSGPQGIR	1	2	1	F1LS40		0,0000	0	0,21	46	0,000251	2	868,46379	0,23	14,77	0
	High	GVVGPQGAR	2	2	1	F1LS40		0,0000	0	0,24	39	0,00134	2	840,46861	-0,08	15,30	0
F1M6Q3	Protein Col4a2 OS=Rattus norvegicus GN=Col4a2 PE=4 SV=2 - [F1M6Q3_RAT]	165,72	5,38	1	6	6	6	1690	166,1	8,65						
	A2	Sequence	# PSMs	# Proteins	# Protein Groups	Protein Group Accessions	Modifications	ΔCn	q-Value	PEP	IonScore	Exp Value	Charge	MH+ [Da]	ΔM [ppm]	RT [min]	# Missed Cleavages
	High	GLDGFQGPSGPR	1	1	1	F1M6Q3		0,0000	0	0,07	69	1,12E-06	2	1187,58147	0,87	21,25	0
	High	LWSGYSLLYFEGQEK	1	1	1	F1M6Q3		0,0000	0	0,16	61	9,39E-06	2	1819,89031	0,07	35,35	0
	High	SVSIGYLLVK	1	1	1	F1M6Q3		0,0000	0	0,12	58	1,93E-06	2	1078,65056	-0,14	30,14	0
	High	AHNQDLGLAGScLAR	1	1	1	F1M6Q3	C12(Carbamidomethyl)	0,0000	0	0,39	30	0,008916	3	1582,77390	-1,03	20,72	0
	High	IAVQPGTmGPQGR	1	1	1	F1M6Q3	M8(Oxidation)	0,0000	0	0,35	27	0,019706	2	1327,67888	0,08	16,81	0
	High	SYWLSTTAPLPmmPVAEEEIKPYISR	1	1	1	F1M6Q3	M12(Oxidation); M13(Oxidation)	0,0000	0	0,36	12	0,270528	3	3041,50284	0,94	33,64	0

P02770	Serum albumin OS=Rattus norvegicus GN=Alb PE=1 SV=2 - [ALBU_RAT]	159,18	4,93	1	4	4	6	608	68,7	6,48						
	A2	Sequence	# PSMs	# Proteins	# Protein Groups	Protein Group Accessions	Modifications	ΔCn	q-Value	PEP	IonScore	Exp Value	Charge	MH+ [Da]	ΔM [ppm]	RT [min]	# Missed Cleavages
	High	EccHGDLLEcADDR	2	1	1	P02770	C2(Carbamidomethyl); C3(Carbamidomethyl); C10(Carbamidomethyl)	0,0000	0	0,02	79	1,3E-08	2	1749,66460	1,13	19,30	0
	High	LcVLHEK	2	1	1	P02770	C2(Carbamidomethyl)	0,0000	0	0,32	36	0,002266	2	898,48137	-0,18	16,81	0
	High	TPVSEKVTK	1	1	1	P02770		0,0000	0	0,34	35	0,002245	3	988,56772	0,34	13,03	1
	High	LcVLHEKTPVSEK	1	1	1	P02770	C2(Carbamidomethyl)	0,0000	0	0,32	25	0,025259	3	1539,82083	0,54	17,74	1
D4A115	Protein Col6a3 OS=Rattus norvegicus GN=Col6a3 PE=4 SV=2 - [D4A115_RAT]	161,83	1,67	2	2	2	3	2211	240,0	6,38						
	A2	Sequence	# PSMs	# Proteins	# Protein Groups	Protein Group Accessions	Modifications	ΔCn	q-Value	PEP	IonScore	Exp Value	Charge	MH+ [Da]	ΔM [ppm]	RT [min]	# Missed Cleavages
	High	SALLDSIQNLQVALTSK	2	2	1	D4A115		0,0000	0	0,25	93	1,65E-09	2	1801,01055	2,17	38,57	0
	High	LNLLDLDYELAEQLDNIAEK	1	2	1	D4A115		0,0000	0	0,46	17	0,13875	3	2332,19162	-0,14	45,45	0
F1LPD0	Protein Col15a1 (Fragment) OS=Rattus norvegicus GN=Col15a1 PE=1 SV=2 - [F1LPD0_RAT]	149,06	5,70	1	5	5	5	1334	137,0	4,91						

	A2	Sequence	# PSMs	# Proteins	# Protein Groups	Protein Group Accessions	Modifications	ΔCn	q-Value	PEP	IonScore	Exp Value	Charge	MH+ [Da]	ΔM [ppm]	RT [min]	# Missed Cleavages
	High	NLVTALSDmGDmLQK	1	1	1	F1LPD0	M9(Oxidation); M12(Oxidation)	0,0000	0	0,16	96	2,39E-09	2	1667,79924	0,76	27,53	0
	High	SSRPLTFEPSAGIFVGNAGATGLER	1	1	1	F1LPD0		0,0000	0	0,03	54	1,56E-05	3	2534,30326	1,32	30,60	0
	High	VVWHGSNPHGVR	1	1	1	F1LPD0		0,0000	0	0,18	28	0,017313	4	1344,69289	0,64	14,62	0
	High	FSLPIVNLK	1	1	1	F1LPD0		0,0000	0	0,43	26	0,00578	2	1030,63017	0,57	32,47	0
	High	FTGSIQQLIIYSDPR	1	1	1	F1LPD0		0,0000	0	0,4	22	0,054893	3	1737,91770	0,36	32,97	0
F1M0B2	Protein LOC683295 (Fragment) OS=Rattus norvegicus GN=LOC683295 PE=4 SV=2 - [F1M0B2_RAT]	151,87	4,11	2	2	2	2	535	57,7	7,88							
	A2	Sequence	# PSMs	# Proteins	# Protein Groups	Protein Group Accessions	Modifications	ΔCn	q-Value	PEP	IonScore	Exp Value	Charge	MH+ [Da]	ΔM [ppm]	RT [min]	# Missed Cleavages
	High	SLDLDSIIAEVK	1	2	1	F1M0B2		0,0000	0	0,03	97	1,95E-09	2	1302,71538	0,14	36,03	0
	High	YEELQITAGR	1	2	1	F1M0B2		0,0000	0	0,04	78	1,79E-07	2	1179,60124	0,65	21,61	0
G3V763	Collagen alpha-1(V) chain OS=Rattus norvegicus GN=Col5a1 PE=4 SV=2 - [G3V763_RAT]	135,61	2,44	2	2	2	3	1636	161,7	4,83							
	A2	Sequence	# PSMs	# Proteins	# Protein Groups	Protein Group Accessions	Modifications	ΔCn	q-Value	PEP	IonScore	Exp Value	Charge	MH+ [Da]	ΔM [ppm]	RT [min]	# Missed Cleavages
	High	GPmVSAQESQAQAILQQAR	2	2	1	G3V763	M3(Oxidation)	0,0000	0	0,07	89	1,22E-08	2	2029,02324	4,94	25,86	0

	High	ILDDEIFEG DIQQLLFVS DHR	1	2	1	G3V763		0,0000	0	0,45	35	0,00222	3	2502,2616 3	4,16	41,27	0
Q6I FW6	Keratin, type I cytoskelet al 10 OS=Rattus norvegicus GN=Krt10 PE=3 SV=1 - [K1C10_R AT]	116,13	7,98	1	5	5	5	526	56,5	5,15							
	A2	Sequence	# PSMs	# Proteins	# Protein Groups	Protein Group Accessions	Modificatio ns	ΔCn	q-Value	PEP	IonScore	Exp Value	Charge	MH+ [Da]	ΔM [ppm]	RT [min]	# Missed Cleavages
	High	QSLEASLAE TEGR	1	1	1	Q6IFW6		0,0000	0	0,17	99	1,32E-09	2	1390,6818 1	0,63	22,76	0
	High	LKYENEVAL R	1	1	1	Q6IFW6		0,0000	0	0,41	31	0,008502	3	1234,6798 7	0,66	19,10	1
	High	LAADDFR	1	1	1	Q6IFW6		0,0000	0	0,29	24	0,057384	2	807,39958	-0,01	18,66	0
	High	QSVEADIN GLR	1	1	1	Q6IFW6		0,0000	0	0,41	23	0,045462	2	1201,6185 8	1,15	22,70	0
	High	QSVEADIN GLRR	1	1	1	Q6IFW6		0,0000	0	0,29	19	0,163225	3	1357,7193 3	0,75	20,66	1
Q6I MF3	Keratin, type II cytoskelet al 1 OS=Rattus norvegicus GN=Krt1 PE=2 SV=1 - [K2C1_RA T]	114,80	5,76	1	3	3	3	625	64,8	7,87							
	A2	Sequence	# PSMs	# Proteins	# Protein Groups	Protein Group Accessions	Modificatio ns	ΔCn	q-Value	PEP	IonScore	Exp Value	Charge	MH+ [Da]	ΔM [ppm]	RT [min]	# Missed Cleavages
	High	FLEQQNQV LQTK	1	1	1	Q6IMF3		0,0000	0	0,12	65	3,34E-06	2	1475,7872 8	1,33	21,71	0
	High	WELLQQVD TSTR	1	1	1	Q6IMF3		0,0000	0	0,06	63	6,21E-06	2	1475,7506 6	1,16	29,02	0
	High	TNAENEFV TIKK	1	1	1	Q6IMF3		0,0000	0	0,24	25	0,027418	3	1393,7291 3	-2,23	19,13	1
Q9 WV H8	Fibulin-5 OS=Rattus norvegicus GN=Fbln5 PE=2 SV=1 -	86,76	4,46	1	2	2	3	448	50,1	4,67							

	[FBLN5_RAT]																
	A2	Sequence	# PSMs	# Proteins	# Protein Groups	Protein Group Accessions	Modifications	ΔCn	q-Value	PEP	IonScore	Exp Value	Charge	MH+ [Da]	ΔM [ppm]	RT [min]	# Missed Cleavages
	High	YPGAYYIFQIK	2	1	1	Q9WVH8		0,0000	0	0,09	52	6,13E-05	2	1362,70976	0,36	32,45	0
	High	DQPFTILFR	1	1	1	Q9WVH8		0,0000	0	0,46	29	0,012694	2	1136,60979	-0,12	34,57	0
F1LR02	Procollagen, type XVIII, alpha 1, isoform CRA_a OS=Rattus norvegicus GN=Col18a1 PE=4 SV=2 - [F1LR02_RAT]	72,57	3,43	1	2	2	2	1311	134,6	6,29							
	A2	Sequence	# PSMs	# Proteins	# Protein Groups	Protein Group Accessions	Modifications	ΔCn	q-Value	PEP	IonScore	Exp Value	Charge	MH+ [Da]	ΔM [ppm]	RT [min]	# Missed Cleavages
	High	ISQVEDPHVGLAYVFGPYSK	1	1	1	F1LR02		0,0000	0	0,06	51	6,49E-05	3	2206,11838	0,19	32,22	0
	High	GAGLFVGQAGAADPDKFQGmISELR	1	1	1	F1LR02	M20(Oxidation)	0,0000	0	0,12	44	0,000388	3	2551,26505	1,55	30,45	1
F1LST1	Fibronectin OS=Rattus norvegicus GN=Fn1 PE=4 SV=2 - [F1LST1_RAT]	85,80	3,14	6	3	3	4	1848	202,4	5,39							
	A2	Sequence	# PSMs	# Proteins	# Protein Groups	Protein Group Accessions	Modifications	ΔCn	q-Value	PEP	IonScore	Exp Value	Charge	MH+ [Da]	ΔM [ppm]	RT [min]	# Missed Cleavages
	High	NTFAEVTGLSPGVTYLFK	2	6	1	F1LST1		0,0000	0	0,13	59	9,69E-06	2	1944,01323	0,95	36,71	0
	High	LGVRPSQGGEAPR	1	6	1	F1LST1		0,0000	0	0,23	21	0,055695	3	1323,71234	-0,38	14,78	0

	High	TGLDSPTG FDSSDVTA NSFTVHWV APR	1	6	1	F1LST1		0,0000	0	0,45	10	0,601645	3	2864,3446 4	-1,42	32,99	0
F1L NH3	Procollage n, type VI, alpha 2, isoform CRA_a OS=Rattus norvegicus GN=Col6a 2 PE=4 SV=2 - [F1LNH3_ RAT]	34,71	2,53	1	2	2	2	1027	109,6	6,61							
	A2	Sequence	# PSMs	# Proteins	# Protein Groups	Protein Group Accessions	Modificatio ns	ΔCn	q-Value	PEP	IonScore	Exp Value	Charge	MH+ [Da]	ΔM [ppm]	RT [min]	# Missed Cleavages
	High	VFAVVITD GR	1	1	1	F1LNH3		0,0000	0	0,31	35	0,002366	2	1076,6099 1	-0,02	25,31	0
	High	VGVVQYSH EGTFEAIR	1	1	1	F1LNH3		0,0000	0	0,37	17	0,186818	3	1791,9017 7	-0,40	23,54	0
D4A 9H2	Dermatop ontin (Predicted), isoform CRA_c OS=Rattus norvegicus GN=Dpt PE=4 SV=1 - [D4A9H2_ RAT]	54,82	18,07	2	2	2	2	166	20,0	5,15							
	A2	Sequence	# PSMs	# Proteins	# Protein Groups	Protein Group Accessions	Modificatio ns	ΔCn	q-Value	PEP	IonScore	Exp Value	Charge	MH+ [Da]	ΔM [ppm]	RT [min]	# Missed Cleavages
	High	cSNNGLVA GFQSR	1	2	1	D4A9H2	C1(Carbami domethyl)	0,0000	0	0,14	41	0,000712	2	1409,6592 3	0,10	21,55	0
	High	QGFSYQcP HGQVVVAV R	1	2	1	D4A9H2	C7(Carbami domethyl)	0,0000	0	0,28	34	0,00413	3	1931,9553 0	0,39	23,38	0

Legend: Protein ID: protein identifier; PSM: peptide-spectrum matching; MW: molecular weight; Calc. pI: calculated isoelectric point.
